# Changes in observed bloodstream infection epidemiology during a single-set blood culture restriction: an interrupted time series analysis

**DOI:** 10.1128/jcm.00296-26

**Published:** 2026-09-25

**Authors:** Joseph B. Ladines-Lim, Bailey Van, Leigh Cressman, Warren B. Bilker, Kathleen O. Degnan, Michael Z. David

**Affiliations:** 1 Division of Infectious Diseases, Department of Medicine, Penn Medicine, Perelman School of Medicine, University of Pennsylvania 6572, Philadelphia, Pennsylvania, USA; 2 Leonard Davis Institute of Health Economics, University of Pennsylvania 6572, Philadelphia, Pennsylvania, USA; 3 Center for Clinical Epidemiology and Biostatistics, Perelman School of Medicine, University of Pennsylvania 6572, Philadelphia, Pennsylvania, USA; 4 Fu Foundation School of Engineering and Applied Science, Columbia University 5798, New York, New York, USA; 5 Department of Biostatistics, Epidemiology, amd Informatics, Perelman School of Medicine, University of Pennsylvania 6572, Philadelphia, Pennsylvania, USA; Maine Medical Center Department of Medicine, Portland, Maine, USA

**Keywords:** blood cultures, diagnostic stewardship, supply shortage, bloodstream infections, epidemiology

## Abstract

During the 2024 US national blood culture bottle shortage, many hospitals implemented restrictions limiting blood culture collection to conserve supplies. The impact of these constraints on the microbiologic epidemiology of bloodstream infections (BSIs) remains incompletely characterized. We conducted an interrupted time series analysis of BSI episodes identified at two quaternary hospitals between June 2023 and June 2025, encompassing a systemwide restriction limiting clinicians to one blood culture set per patient every 24 h from June through December 2024. We grouped blood cultures into discrete infection episodes and categorized these by organism group. We normalized monthly incidence rates to acute-care patient-days and used segmented Poisson regression models to estimate changes in incidence associated with restriction onset and withdrawal. We identified 5,391 BSI episodes among 4,484 patients. During the restriction period, the relative proportion of episodes due to enterococci, fungi, gram-negative rods, and *Staphylococcus aureus*/*lugdunensis* increased, whereas episodes due to anaerobes, streptococci, coagulase-negative staphylococci/common commensals, and polymicrobial infections decreased. Interrupted time series analyses demonstrated an overall decline in detected BSI incidence, with statistically significant decreases observed for streptococci and gram-negative rods. Estimates for other groups were limited by small numbers and imprecision. Blood culture restrictions during a national supply shortage were associated with measurable shifts in the detected distribution of bloodstream pathogens and reduced overall BSI detection. These findings suggest that diagnostic constraints may selectively impair detection of certain organism groups, with important implications for laboratory surveillance, antimicrobial stewardship, and interpretation of microbiologic trends during periods of constrained testing.

**IMPORTANCE:**

Blood cultures remain the primary method for diagnosing bloodstream infections and tracking causative pathogens. During the 2024 national blood culture bottle shortage, many hospitals limited blood culture collection to conserve supplies. While prior studies focused on changes in testing volume or clinical outcomes, it has remained unclear how these restrictions affected which organisms were ultimately detected. Here, we examined how the shortage influenced the observed distribution of major bloodstream pathogens, including *Staphylococcus aureus/lugdunensis*, gram-negative rods, streptococci, enterococci, anaerobes, fungi, and coagulase-negative staphylococci/common commensals. Restricting blood culture collection was associated with a decline in overall detected bloodstream infections and shifts in the relative frequency of specific organism groups. These findings suggest that limitations in diagnostic testing can distort microbiologic surveillance data and alter the apparent epidemiology of bloodstream pathogens. Laboratories, clinicians, and surveillance systems should consider how supply disruptions and subsequent testing policies may influence the organisms identified in clinical practice.

## INTRODUCTION

Although novel nucleic acid-based platforms have expanded in recent years, blood cultures remain the primary diagnostic test for bloodstream infections (BSIs) and the foundation of microbiologic surveillance in hospitalized patients. Clinical guidelines generally recommend collection of multiple culture sets to optimize diagnostic sensitivity and interpretability (1).

During the 2024 US national shortage of Becton Dickinson (BD) BACTEC blood culture bottles (2), many hospitals implemented ordering restrictions to conserve supplies (3–12). Prior studies have primarily examined the impact of these restrictions on blood culture utilization and clinical outcomes, with mixed findings (3, 4, 6, 8–12). Data from our own institution have suggested that shortage-related restrictions were associated with decreased blood culture ordering, delays in diagnostic testing and antimicrobial administration, and potentially adverse shifts in in-hospital mortality trends, albeit with causal inference limited by our study’s observational design (13). However, these analyses did not evaluate organism-specific culture results.

Determining whether diagnostic constraints disproportionately affect detection of specific organism groups across BSIs of any etiology is critical for interpreting microbiologic surveillance data, antibiograms, antimicrobial stewardship metrics, and quality benchmarks. To date, few studies have examined organism-level effects of the shortage, with existing analyses largely focusing on selected pathogens (4, 7).

To address this knowledge gap, we conducted an interrupted time series analysis of all BSIs (13) categorized by organism group before, during, and after implementation of blood culture restrictions during the national shortage. Building on our prior institutional analyses (13), this study evaluates how diagnostic constraints influenced the detected epidemiology of BSIs across major bacterial and fungal pathogen groups.

## MATERIALS AND METHODS

We performed an interrupted time series analysis of BSI episodes identified at the Hospital of the University of Pennsylvania (1,069 beds) and Penn Presbyterian Medical Center (347 beds) between 26 June 2023 and 25 June 2025, encompassing a systemwide electronic order restriction implemented 26 June–23 December 2024, that limited clinicians to one blood culture set per patient every 24 hours. A blood culture set was defined as one aerobic and one anaerobic bottle, collected and ordered together as a single unit at our institution; aerobic-only or anaerobic-only orders were exceedingly rare throughout the study period. Accompanying guidance during the restriction period recommended filling each bottle with a minimum of 10 mL of blood to optimize sensitivity, and a tip sheet on blood culture collection best practices was distributed to hospital staff; however, blood volume per bottle was not captured in the data sources used for this analysis.

We extracted data from Epic Clarity (Epic Systems, Verona, WI, USA) and TheraDoc (Premier, Inc., Charlotte, NC, USA), including encounters for inpatient hospitalizations, medical observations, and emergency department visits and associated blood culture testing. We deduplicated and grouped blood cultures into discrete bloodstream infection episodes, rather than examining each culture, using prespecified temporal and microbiologic criteria (see Methods S1). We categorized episodes into the following primary organism groups: gram-negative rods, *Staphylococcus aureus/lugdunensis*, streptococci, enterococci, anaerobes, fungi, coagulase-negative staphylococci (CoNS)/common commensals, other rare individual organisms, and polymicrobial infections (see Methods S1 and S2 and Table S1). We considered episodes polymicrobial only if there were organisms from >1 primary organism group except coagulase-negative staphylococci/common commensals or the rare organisms group; if there were multiple organisms from the same organism group, then we classified the episode accordingly (e.g., we classified a BSI episode with both *Streptococcus anginosus* and *Streptococcus pyogenes* as part of the “streptococci” group). We normalized monthly episode incidence rates by acute-care patient-days, i.e., the total amount of time within all encounters (inpatient hospitalizations, medical observations, and emergency department visits) for that time interval. We did not use International Classification of Diseases (ICD)-based diagnosis codes to characterize illness severity or infection source, given known limitations in the sensitivity and specificity of ICD coding for syndromes such as sepsis (14); instead, we relied on the Elixhauser Comorbidity Index (15), which was largely similar across study periods (Table S2).

We fit segmented Poisson regression models to estimate incidence rate ratios (IRRs) for level and slope changes associated with restriction onset and withdrawal, with Newey-West robust standard errors (16) and lag after each change point and maximum lag length guided by the Cumby-Huizinga test (17). We performed analyses for all episodes combined and stratified by organism group.

We employed two-sided hypothesis tests with *α* = 0.05 and conducted all analyses using R version 4.3.0 (R Foundation for Statistical Computing, Vienna, Austria) (18). We followed the Strengthening the Reporting of Observational Studies in Epidemiology guideline (19).

## RESULTS

We identified a total of 5,391 BSI episodes among 4,484 unique patients during the 2-year study period overall, with baseline patient characteristics stable across study periods (Table S2). Organism group distribution, blood culture utilization metrics, and select hospital outcomes varied by study period (Table 1), as did counts of individual organisms (Tables S3 to S11).

**TABLE 1:** Organism group epidemiology, hospital outcomes, blood culture outcomes, and antimicrobial outcomes of bloodstream infection episodes aggregated by time period^*a*^^,^^*b*^

Outcome	Pre-restriction (*N* = 2,987; acute-care patient-days = 511,592)	Restriction (*N* = 1,076; acute-care patient-days = 262,130)	Post-restriction (*N* = 1,328; acute-care patient-days = 279,384)	Overall (*N* = 5,391; acute-care patient-days = 1,053,106)
Organism group, No. (**%**)				
Anaerobes	132 (4.4)	34 (3.2)	61 (4.6)	227 (4.2)
Enterococci	156 (5.2)	79 (7.3)	89 (6.7)	324 (6.0)
Fungi	118 (4.0)	62 (5.8)	53 (4.0)	233 (4.3)
Gram-negative rods	761 (25.5)	323 (30.0)	366 (27.6)	1,450 (26.9)
*Staphylococcus aureus* or *lugdunensis*	336 (11.2)	159 (14.8)	158 (11.9)	653 (12.1)
Streptococci	301 (10.1)	82 (7.6)	123 (9.3)	506 (9.4)
Coagulase-negative staphylococci or common commensals	1,010 (33.8)	297 (27.6)	392 (29.5)	1,699 (31.5)
Polymicrobial	152 (5.1)	34 (3.2)	83 (6.2)	269 (5.0)
Rare organisms	21 (0.7)	6 (0.6)	3 (0.2)	30 (0.6)
Hospital outcomes				
In-hospital mortality or hospice discharge, No. (%)	344 (11.5)	96 (8.9)	126 (9.5)	566 (10.5)
Total 30-day revisits, mean (SD) and median [IQR]	0.34 (0.91) 0.00 [0.00, 0.00]	0.37 (0.73) 0.00 [0.00, 1.00]	0.35 (1.00) 0.00 [0.00, 0.00]	0.35 (0.90) 0.00 [0.00, 0.00]
Total number of encounters, mean (SD) and median [IQR]	1.12 (0.70) 1.00 [1.00, 1.00]	1.08 (0.50) 1.00 [1.00, 1.00]	1.05 (0.34) 1.00 [1.00, 1.00]	1.10 (0.59) 1.00 [1.00, 1.00]
Total length of stay, days, mean (SD) and median [IQR]	22.59 (31.13) 11.32 [5.41, 25.71]	25.73 (35.35) 13.75 [6.33, 30.69]	23.28 (28.78) 12.95 [6.41, 29.13]	23.39 (31.49) 12.04 [5.84, 27.33]
Blood culture outcomes				
Total number of blood cultures, mean (SD) and median [IQR]	5.44 (5.47) 4.00 [3.00, 6.00]	2.66 (3.30) 2.00 [2.00, 3.00]	4.62 (3.94) 4.00 [2.00, 6.00]	4.69 (4.87) 4.00 [2.00, 5.00]
Total number of blood cultures positive for main organism, mean (SD) and median [IQR]	2.00 (2.37) 1.00 [1.00, 2.00]	1.34 (1.07) 1.00 [1.00, 1.00]	1.89 (1.64) 1.00 [1.00, 2.00]	1.84 (2.02) 1.00 [1.00, 2.00]
Number of calendar days with ≥1 blood culture positive for main organism obtained, mean (SD) and median [IQR]	1.40 (1.40) 1.00 [1.00, 1.00]	1.27 (0.87) 1.00 [1.00, 1.00]	1.31 (0.92) 1.00 [1.00, 1.00]	1.35 (1.20) 1.00 [1.00, 1.00]
Time to first blood culture, days, mean (SD) and median [IQR]	5.98 (15.07) 0.12 [0.03, 4.24]	7.99 (20.24) 0.23 [0.05, 6.81]	7.73 (18.20) 0.20 [0.05, 7.41]	6.81 (17.03) 0.16 [0.04, 5.56]
Time to first blood culture positive for main organism, days, mean (SD) and median [IQR]	6.61 (19.00) 0.15 [0.04, 4.59]	8.16 (21.05) 0.23 [0.05, 7.09]	7.81 (18.25) 0.21 [0.05, 7.52]	7.22 (19.26) 0.18 [0.04, 5.56]
≥1 probable contaminant organism, No. (%)	1,175 (39.3)	331 (30.8)	475 (35.8)	1,981 (36.7)
Number of distinct organisms found on culture, mean (SD) and median [IQR]	1.21 (0.63) 1.00 [1.00, 1.00]	1.13 (0.52) 1.00 [1.00, 1.00]	1.24 (0.63) 1.00 [1.00, 1.00]	1.20 (0.61) 1.00 [1.00, 1.00]
Clearance of infection documented on culture, No. (%)	2,554 (85.5)	749 (69.6)	1,086 (81.8)	4,389 (81.4)

^*a*^ The pre-restriction, restriction, and post-restriction periods were 26 June 2023–25 June 2024, 26 June 2024–23 December 2024, and 24 December 2024–25 June 2025, respectively. For categorical variables, data shown are the number of bloodstream infectious episodes having the indicated outcome, with percentage in parentheses reflecting this as the numerator and size of the overall group as the denominator (shown in the headings). For continuous or count variables, data shown are the mean number of episodes with the indicated outcome and standard deviation in parentheses, as well as the median and interquartile range in brackets. The headings also show the total number of acute-care patient-days for each period, as we used this as the denominator for normalizing organism group incidence rates in interrupted time series analyses. Total 30-day revisits and total number of encounters in hospital outcomes included inpatient hospitalizations, emergency department visits, and medical observation visits, which was also the case for acute-care patient-days calculated for each time period. Any blood culture outcomes pertaining to the main organism refer to the primary organism representative of the organism group that the episode was categorized as (e.g., *Escherichia coli *representative of gram-negative rods).

^*b*^ IQR, interquartile range; SD, standard deviation.

**Fig 1:**
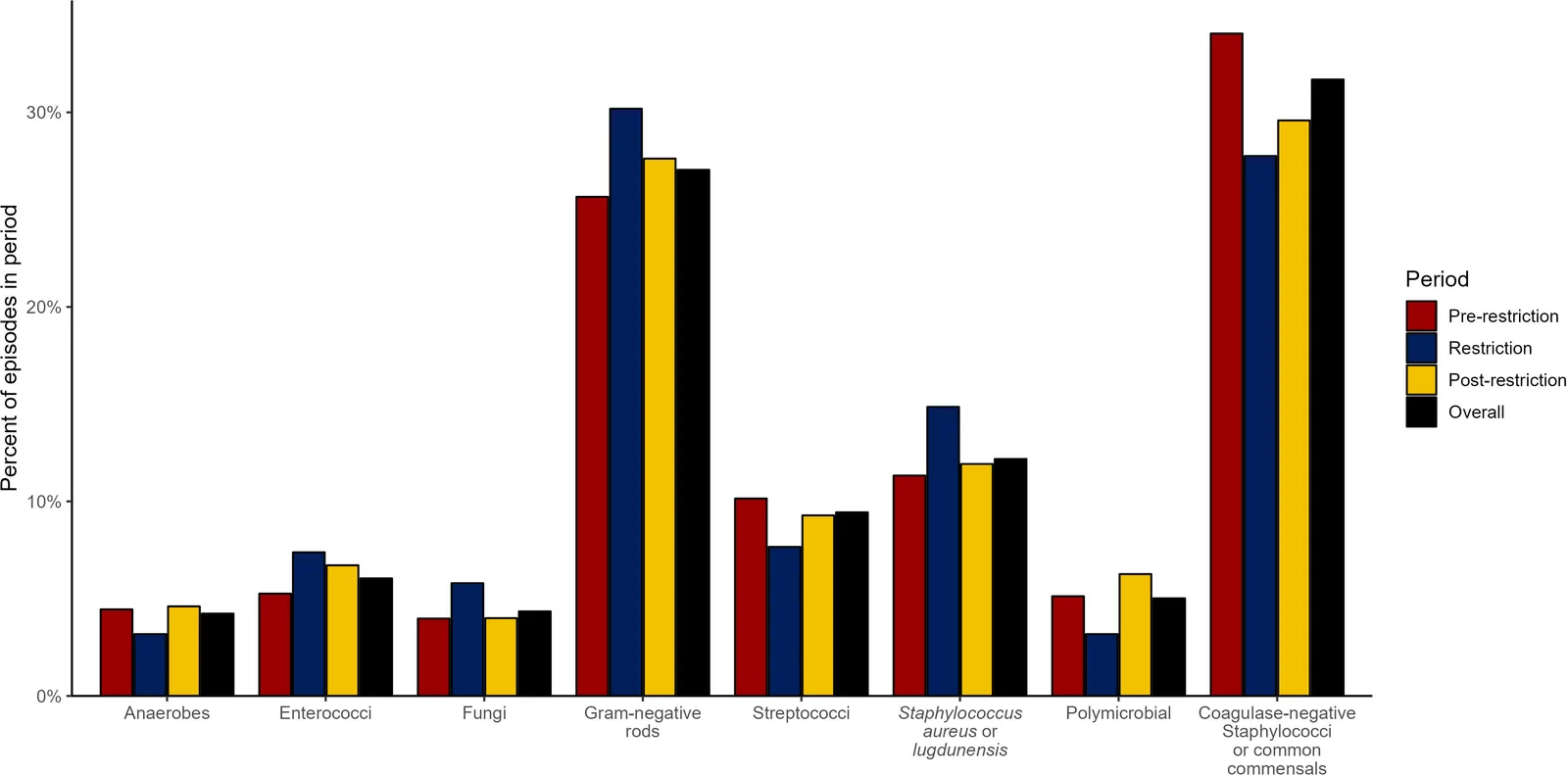
Proportion of organism groups among all bloodstream infection episodes, overall and by pre-restriction, restriction, and post-restriction time period. The pre-restriction, restriction, and post-restriction periods were 26 June 2023–25 June 2024, 26 June 2024–23 December 2024, and 24 December 2024–25 June 2025, respectively. We omitted showing the “Rare organism” group due to its overall rare incidence, <1% across all time periods.

**Fig 2:**
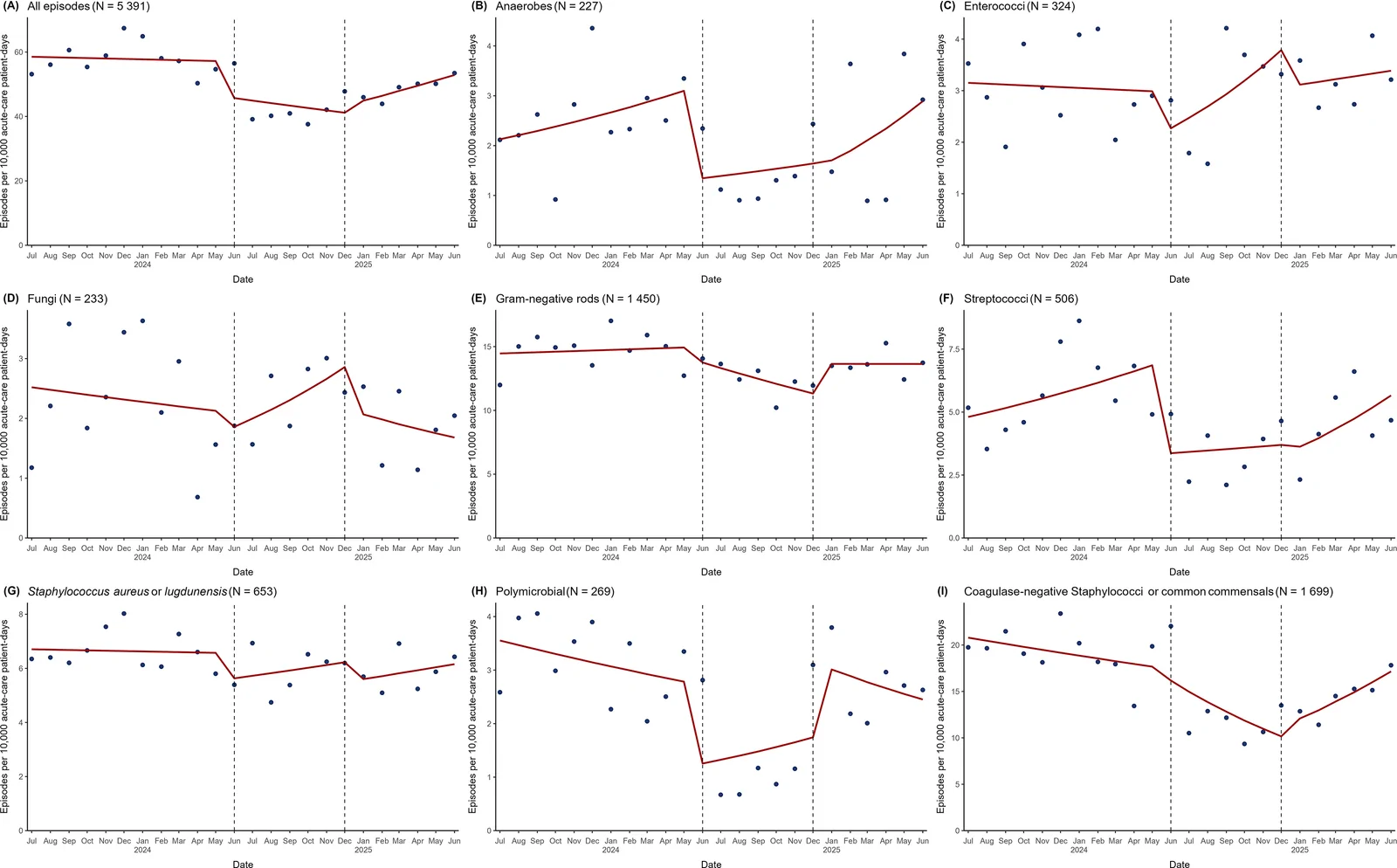
Interrupted time series of blood culture incidence at both hospital sites combined, indicating three study periods, all episodes and by organism group, 26 June 2023–25 June 2025. Blue dots represent weekly averages, and the red solid line represents the fitted line from the segmented regression model assessing for abrupt level and slope changes at the start and stop dates of the blood culture restriction of one blood culture set per patient per 24 h, which were 26 June 2024 and 23 December 2024, respectively, represented by dashed vertical lines. The figure displays monthly incidence of bloodstream infection episodes for the following groups: (**A**) All episodes. (**B**) Anaerobes. (**C**) Enterococci. (**D**) Fungi. (**E**) Gram-negative rods. (**F**) Streptococci. (**G**) *Staphylococcus aureus* or *lugdunensis.* (**H**) Polymicrobial. (**I**) Coagulase-negative staphylococci or common commensals.

During the restriction period, the relative proportion of episodes due to enterococci, fungi, gram-negative rods, and *S. aureus*/*lugdunensis* increased, while episodes due to anaerobes, streptococci, coagulase-negative staphylococci/commensals, and polymicrobial infections decreased (Fig. 1). Interrupted time series analyses demonstrated a decrease in overall BSI incidence during the restriction period, with declines observed for several organism groups, including gram-negative rods, streptococci, coagulase-negative staphylococci/common commensals, and polymicrobial infections (Fig. 2).

We observed statistically significant effects only for decreases in streptococci incidence upon restriction onset (IRR, 0.483; 95% CI, 0.243–0.963; *P* = 0.04) and gram-negative rod incidence during restriction (IRR, 0.965; 95% CI, 0.936–0.995; *P* = 0.02) (Table S12; Fig. 2). Other statistically significant estimates included a pre-restriction increase in anaerobe incidence (IRR, 1.038; 95% CI, 1.010–1.067; *P* = 0.007), an increase in enterococci incidence during restriction (IRR, 1.095; 95% CI, 1.001–1.197; *P* = 0.05), a post-restriction decrease in fungal incidence (IRR, 0.893; 95% CI, 0.810–0.985; *P* = 0.02), a post-restriction increase in gram-negative rod incidence (IRR, 1.033; 95% CI, 1.005–1.061; *P* = 0.02), a decrease in *Staphylococcus aureus* or *lugdunensis* incidence upon restriction withdrawal (IRR, 0.744; 95% CI, 0.590–0.938; *P* = 0.01), and a post-restriction increase in incidence of coagulase-negative staphylococci or common commensals (IRR, 1.159; 95% CI, 1.027–1.308; *P* = 0.02) (Table S12); however, when taken together with visual trends (Fig. 2), these estimates appear artifactual. Other estimates did not show significant change; many comparisons were limited by small numbers of observations despite suggestive visual trends.

Among episodes with available blood culture ordering data, 2,684 of 2,987 (89.9%) in the pre-restriction period had ≥2 blood cultures obtained, compared with 1,155 of 1,328 (87.0%) in the post-restriction period (*χ*^2^ = 7.5, *P* = 0.006), indicating that adherence to recommended multi-set ordering was incomplete at baseline and did not improve following restriction withdrawal.

## DISCUSSION

In this interrupted time series analysis, implementation of a single-set blood culture restriction during the 2024 national supply shortage was associated with measurable changes in the detected epidemiology of BSIs, including an overall decline in BSI incidence and heterogeneous effects across major organism groups. These findings suggest that diagnostic constraints may selectively influence pathogen detection rather than uniformly suppressing identification across infections.

Our results extend prior work evaluating the clinical and operational impact of the blood culture bottle shortage by demonstrating systematic effects on microbiologic surveillance. A prior study on *Staphylococcus aureus* bacteremia found that similar restriction policies were not associated with worsened outcomes in that population, despite reduced culture collection (4). A separate Centers for Disease Control and Prevention-led analysis documented widespread disruptions in blood culture practices and decreased detection of selected pathogens at facilities reliant on BD BACTEC, including *Escherichia coli*, *Staphylococcus aureus*, *Streptococcus pneumoniae*, and *Streptococcus pyogenes* (7). The restriction implemented at our institution was applied uniformly via electronic order restriction, without differential guidance by clinical unit, service, or patient risk category. The prior analysis at our institution found a significant decline in the proportion of hospitalizations with any blood culture ordered during the restriction period, alongside implementation of a post-restriction order panel to standardize appropriate blood culture ordering (13). Additionally, in contrast to previously reported pathogen-specific or utilization-focused analyses, our study leveraged comprehensive episode-level data across all major organism groups, demonstrating that restriction policies may alter the apparent distribution of bloodstream pathogens in addition to reducing overall detection.

The relative increase in enterococci, gram-negative rods, fungi, and *Staphylococcus aureus/lugdunensis* during the restriction period likely reflects preferential detection of pathogens associated with higher organism burden, greater illness severity, or persistent infection. Such infections may remain detectable despite limited sampling. Conversely, decreased detection of streptococci, anaerobes, commensal organisms, and polymicrobial infections likely reflects reduced sensitivity for lower-grade, intermittent, or complex infections when repeated sampling is constrained. Reduced opportunities for contamination may also partially explain declines in commensal organisms. This limitation is particularly relevant for coagulase-negative staphylococci, where distinguishing contamination from true bacteremia typically relies on growth in multiple sets or careful clinical correlation. During the restriction, clinicians had only one set per 24 h available to make this determination, which may have made adjudication more difficult and could have increased pressure to treat isolated CoNS empirically as true infection rather than probable contamination.

These microbiologic findings also complement our prior work demonstrating reduced blood culture ordering and delayed diagnostics during the restriction period (13). Together, these data suggest that constrained testing affected both whether cultures were obtained and which pathogens were ultimately detected, contributing to the observed decline in overall detected BSI incidence. Such effects may help contextualize previously observed trends in clinical outcomes, including potential adverse shifts in in-hospital mortality trends, by suggesting reduced sensitivity for timely identification of certain BSIs.

Interpretation of organism-specific interrupted time series analyses was limited in some strata by low event counts and increased variability. While select estimates achieved statistical significance and were concordant with visual trends, others appeared inconsistent and likely reflected model instability. These findings underscore the importance of integrating graphical and statistical approaches when evaluating interrupted time series data in laboratory surveillance studies, particularly when event rates are low.

Our study has several limitations. First, this was an observational analysis conducted within a single health system, limiting causal inference and generalizability. In particular, our cohort reflects the case mix of two quaternary academic referral hospitals with a high burden of comorbidity (Elixhauser Comorbidity Index ≥30 in >40% of episodes across all periods; Table S2) and a substantial proportion of hospital-onset infection, which likely explains the long overall length of stay, consistent with prior literature (20) and underscoring our study’s limited generalizability to lower acuity settings. Second, despite normalization to acute-care patient-days, changes in patient mix, illness severity, or admission patterns during the study period may have influenced observed trends. Third, episode definitions and organism groupings, while prespecified and clinically grounded, remain subject to misclassification. Fourth, altered clinician behavior during the restriction period, including decisions to forgo blood culture collection (13), further complicates interpretation of findings, as observed changes likely reflect both diagnostic constraints and shifts in ordering practices. Fifth, our episode definitions could not adjudicate the clinical significance of individual CoNS isolates, and we did not perform chart review to distinguish contamination from true bacteremia at the episode level; this is a particular limitation during the restriction period, when a single culture set limited the ability to make this distinction and may have increased unnecessary antimicrobial exposure for isolates of uncertain significance. Sixth, we lacked data on blood volume collected per bottle, a key determinant of culture sensitivity; we cannot exclude the possibility that clinicians compensated for the restriction by more completely filling the single available set, which could partially explain the modest overall change in detected BSI incidence. Still, it is unlikely that this may have been mediated through an increased volume fill of a single bottle within the set, given that aerobic and anaerobic bottles are ordered and collected together as a single set at our institution almost universally, with isolated single-bottle orders exceedingly rare. Seventh, our data sources did not distinguish blood cultures ordered in the emergency department from those ordered on inpatient units, precluding evaluation of effects specific to care settings; our prior analysis found an increased time to first blood culture during the restriction period, consistent with delayed rather than purely relocated testing (13).

Despite these limitations, our findings offer several lessons for institutions facing future diagnostic supply disruptions. First, blanket restrictions imposed without accompanying diagnostic stewardship infrastructure may have uneven effects on pathogen detection, underscoring the value of pairing supply-driven restrictions with clinical decision support that helps clinicians prioritize testing for patients at highest risk of BSI, an approach described in prior diagnostic stewardship frameworks for blood culture utilization (1, 21). Second, our finding that adherence to recommended multi-set ordering remained incomplete even after restriction withdrawal (89.9% versus 87.0% of episodes with ≥2 cultures pre- and post-restriction, respectively) suggests that education and order-panel interventions alone may be insufficient to restore optimal practice, and that structural or default-order-set changes embedded in the electronic health record may be needed. Third, our results show that diagnostic supply disruptions and restrictive testing policies may substantially distort surveillance data, antibiograms, and observed epidemiologic patterns, with downstream consequences for quality metrics and benchmarking that health systems should account for. Finally, our experience highlights the vulnerability of a single-supplier model for a diagnostic test as central to sepsis and BSI management as blood cultures; institutions and health systems may benefit from maintaining relationships with multiple blood culture media suppliers or platforms to reduce dependence on any single manufacturer during future shortages.

## Data Availability

Data are not publicly available. De-identified data and analytic code are available upon reasonable request, subject to institutional policies.

## SUPPLEMENTAL MATERIAL

Supplemental material jcm.00296-26-s0001.docx Supplemental methods and Tables S1 to S12.

ASM does not own the copyrights to Supplemental Material that may be linked to, or accessed through, an article. The authors have granted ASM a non-exclusive, world-wide license to publish the Supplemental Material files. Please contact the corresponding author directly for reuse.
