# Supplementary material for: Changes in observed bloodstream infection epidemiology during a single-set blood culture restriction: an interrupted time series analysis
Source: J Clin Microbiol. 2026 Sep 25;64(10):e00296-26. doi: 10.1128/jcm.00296-26 (PMC13712782; doi:10.1128/jcm.00296-26)
Supplement: Supplementary file 1 [file jcm.00296-26-s0001.docx]

# **Supplement**

**Changes in Observed Bloodstream Infection Epidemiology During a Single-Set Blood Culture Restriction: An Interrupted Time Series Analysis**

Joseph B. Ladines-Lim, MD, PhD^1,2,3^, Bailey Van^1,4^, Leigh Cressman, MA^3^, Warren B. Bilker, PhD^5^, Kathleen O. Degnan, MD^1^, Michael Z. David, MD, PhD^1,3,5^

**Affiliations:**

^1^ Division of Infectious Diseases, Department of Medicine, Penn Medicine, Perelman School of Medicine, University of Pennsylvania, Philadelphia, Pennsylvania, United States of America

^2^ Leonard Davis Institute of Health Economics, University of Pennsylvania, Philadelphia, Pennsylvania, United States of America

^3^ Center for Clinical Epidemiology and Biostatistics, Perelman School of Medicine, University of Pennsylvania, Philadelphia, Pennsylvania, United States of America

^4^ Fu Foundation School of Engineering and Applied Science, Columbia University, New York City, New York, United States of America

^5^ Department of Biostatistics and Epidemiology, Perelman School of Medicine, University of Pennsylvania, Philadelphia, Pennsylvania, United States of America

**Address correspondence to:** Joseph B. Ladines-Lim, MD, PhD, 3400 Spruce St, 3 Silverstein, Ste E, Philadelphia, Pennsylvania 19104-4238. Email: [joseph.ladines-lim@pennmedicine.upenn.edu](mailto:joseph.ladines-lim@pennmedicine.upenn.edu). Phone: 267-581-2092.

**Date:** July 6, 2026

# **Table of Contents**

**Supplementary Methods 1.** Deduplication and Categorization of Blood Cultures into Bloodstream Infection Episodes and Primary Organism Grouping.

**Supplementary Methods 2.** Categorization of Organisms Isolated from Blood Cultures.

**Supplementary Table 1.** Organisms Isolated from Blood Cultures Mapped to Organism Groups.

**Supplementary Table 2.** Baseline Sociodemographic and Clinical Characteristics of Bloodstream Infection Episodes Aggregated by Time Period.

**Supplementary Table 3.** Frequency of Individual Organisms Categorized as Anaerobes in Bloodstream Infection Episodes Aggregated by Time Period.

**Supplementary Table 4.** Frequency of Individual Organisms Categorized as Enterococci in Bloodstream Infection Episodes Aggregated by Time Period.

**Supplementary Table 5.** Frequency of Individual Organisms Categorized as Fungi in Bloodstream Infection Episodes Aggregated by Time Period.

**Supplementary Table 6.** Frequency of Individual Organisms Categorized as Gram-Negative Rods in Bloodstream Infection Episodes Aggregated by Time Period.

**Supplementary Table 7.** Frequency of Individual Organisms Categorized as *Staphylococcus aureus* or *lugdunensis* in Bloodstream Infection Episodes Aggregated by Time Period.

**Supplementary Table 8.** Frequency of Individual Organisms Categorized as Streptococci in Bloodstream Infection Episodes Aggregated by Time Period.

**Supplementary Table 9.** Frequency of Individual Organisms Categorized as Rare Organisms in Bloodstream Infection Episodes Aggregated by Time Period.

**Supplementary Table 10.** Frequency of Individual Organisms Categorized as Coagulase-Negative Staphylococci or Common Commensals in Bloodstream Infection Episodes Aggregated by Time Period.

**Supplementary Table 11.** Frequency of Individual Organisms Categorized as Polymicrobial Bloodstream Infection Episodes Aggregated by Time Period.

**Supplementary Table 12.** Effect Estimates of Single-Set Blood Culture Restriction on Episode Incidence for Primary Organism Groups.

# **Supplementary Methods 1.** Deduplication and Categorization of Blood Cultures into Bloodstream Infection Episodes and Primary Organism Grouping.

We deduplicated and grouped blood cultures into discrete bloodstream infection (BSI) episodes using prespecified temporal and microbiological criteria. First, we mapped cultures to individual patients. Then, we categorized multiple positive cultures growing the same organism within 5 days as belonging to the same episode. Thus, an episode may have included ≥1 positive blood culture. We included negative cultures if they occurred within 48 hours before the first culture positive for the primary organism or within 72 hours after the last culture positive for the primary organism. We regarded the former as potentially reflecting initial testing in the setting of suspected BSI, and the latter as evidence of documented clearance.

We classified organisms into the following groups: anaerobes, enterococci, gram-negative rods, fungi, *Staphylococcus aureus* or *lugdunensis*, streptococci, coagulase-negative staphylococci or common commensals, and rare organisms (see **Supplementary Methods 2**).

In instances where multiple organisms were isolated within the prespecified time windows, we further classified episodes according to the following hierarchy, intending to balance sensitivity for recurrent infection with specificity for distinct infection events:

1. If all organisms were classified as coagulase-negative staphylococci or common commensals, we left the episode classified accordingly.
2. If cultures yielded one or more organisms from a true pathogen group (anaerobes, enterococci, gram-negative rods, fungi, *Staphylococcus aureus* or *lugdunensis*, or streptococci) and ≥1 organism from the coagulase-negative staphylococci or common commensal group or rare organisms group, we classified the episode according to the true pathogen group. For example, if blood cultures resulted in *Staphylococcus aureus* and *Staphylococcus hominis* within the prespecified time window, we classified the episode as “*Staphylococcus aureus* or *lugdunensis*” with *Staphylococcus hominis* considered a likely contaminant.
3. If ≥1 organism from distinct true pathogen groups were isolated from the same blood culture set or from different cultures within 24 hours, we classified the episode as polymicrobial.
4. If organisms from distinct true pathogen groups were isolated outside the same culture set and more than 24 hours apart, we classified the later isolate as a new episode and grouped subsequent cultures accordingly.

# **Supplementary Methods 2.** Categorization of Organisms Isolated from Blood Cultures.

**Supplementary Table 1** lists the classification of organisms isolated from blood cultures into prespecified organism groups. We based organism group assignments on standardized laboratory nomenclature and manual review of uncommon isolates. We classified blood cultures with unspeciated results (e.g., “gram-negative bacilli) as anaerobes if the result specified an anaerobic organism, as gram-negative rods if the result specified a non-anaerobic gram-negative organism, or as coagulase-negative staphylococci or common commensals otherwise.

# **Supplementary Table 1.** Organisms Isolated from Blood Cultures Mapped to Organism Groups.

| **Organism group** | **Organism** |
| --- | --- |
| Anaerobes | *Actinomyces neuii* |
| Anaerobes | Actinomyces species |
| Anaerobes | *Actinotignum schaalii* |
| Anaerobes | *Bacteroides caccae* |
| Anaerobes | *Bacteroides eggerthii* |
| Anaerobes | *Bacteroides fragilis* |
| Anaerobes | *Bacteroides ovatus/xylanisolvens* |
| Anaerobes | *Bacteroides pyogenes* |
| Anaerobes | Bacteroides species |
| Anaerobes | *Bacteroides thetaiotaomicron* |
| Anaerobes | *Bacteroides uniformis* |
| Anaerobes | *Bacteroides vulgatus* |
| Anaerobes | Bifidobacterium species |
| Anaerobes | *Clostridium cadaveris* |
| Anaerobes | *Clostridium clostridioforme* |
| Anaerobes | *Clostridium innocuum* |
| Anaerobes | *Clostridium perfringens* |
| Anaerobes | *Clostridium ramosum* |
| Anaerobes | *Clostridium septicum* |
| Anaerobes | Clostridium species |
| Anaerobes | *Clostridium sporogenes* |
| Anaerobes | *Clostridium tertium* |
| Anaerobes | *Eggerthella lenta* |
| Anaerobes | Eggerthella species |
| Anaerobes | *Finegoldia magna* |
| Anaerobes | *Fusobacterium necrophorum* |
| Anaerobes | *Fusobacterium nucleatum* |
| Anaerobes | *Fusobacterium periodonticum* |
| Anaerobes | Fusobacterium species |
| Anaerobes | *Lacrimispora celerecrescens* |
| Anaerobes | Lancefieldella species |
| Anaerobes | *Paeniclostridium sordellii* |
| Anaerobes | *Parabacteroides distasonis* |
| Anaerobes | *Parvimonas micra* |
| Anaerobes | *Peptoniphilus asaccharolyticus* |
| Anaerobes | Peptoniphilus species |
| Anaerobes | Peptostreptococcus species |
| Anaerobes | *Porphyromonas asaccharolytica* |
| Anaerobes | *Prevotella bivia* |
| Anaerobes | *Prevotella buccae* |
| Anaerobes | *Prevotella intermedia* |
| Anaerobes | *Prevotella melaninogenica* |
| Anaerobes | Prevotella species |
| Anaerobes | *Schaalia odontolyticus* |
| Anaerobes | Tissierella species |
| Anaerobes | *Veillonella dispar* |
| Anaerobes | Veillonella species |
| Anaerobes | *Weissella confusa* |
| Anaerobes | Weissella species |
| Coagulase-negative staphylococci or common commensals | Bacillus species |
| Coagulase-negative staphylococci or common commensals | *Bacillus subtilis* |
| Coagulase-negative staphylococci or common commensals | Brevibacillus species |
| Coagulase-negative staphylococci or common commensals | *Brevibacterium casei* |
| Coagulase-negative staphylococci or common commensals | Brevibacterium species |
| Coagulase-negative staphylococci or common commensals | *Corynebacterium jeikeium* |
| Coagulase-negative staphylococci or common commensals | Corynebacterium species |
| Coagulase-negative staphylococci or common commensals | *Corynebacterium striatum* |
| Coagulase-negative staphylococci or common commensals | *Cutibacterium acnes* |
| Coagulase-negative staphylococci or common commensals | *Cutibacterium avidum* |
| Coagulase-negative staphylococci or common commensals | Dermabacter species |
| Coagulase-negative staphylococci or common commensals | Diphtheroids |
| Coagulase-negative staphylococci or common commensals | Gulosibacter species |
| Coagulase-negative staphylococci or common commensals | *Kocuria rhizophila* |
| Coagulase-negative staphylococci or common commensals | Kocuria species |
| Coagulase-negative staphylococci or common commensals | *Lactobacillus jensenii* |
| Coagulase-negative staphylococci or common commensals | Lactobacillus species |
| Coagulase-negative staphylococci or common commensals | *Micrococcus luteus* |
| Coagulase-negative staphylococci or common commensals | Micrococcus species |
| Coagulase-negative staphylococci or common commensals | Pseudoglutamicibacter species |
| Coagulase-negative staphylococci or common commensals | *Rothia kristinae* |
| Coagulase-negative staphylococci or common commensals | *Rothia mucilaginosa* |
| Coagulase-negative staphylococci or common commensals | Rothia species |
| Coagulase-negative staphylococci or common commensals | *Staphylococcus auricularis* |
| Coagulase-negative staphylococci or common commensals | *Staphylococcus capitis* |
| Coagulase-negative staphylococci or common commensals | *Staphylococcus caprae* |
| Coagulase-negative staphylococci or common commensals | *Staphylococcus cohnii* |
| Coagulase-negative staphylococci or common commensals | *Staphylococcus epidermidis* |
| Coagulase-negative staphylococci or common commensals | *Staphylococcus haemolyticus* |
| Coagulase-negative staphylococci or common commensals | *Staphylococcus hominis* |
| Coagulase-negative staphylococci or common commensals | *Staphylococcus saprophyticus* |
| Coagulase-negative staphylococci or common commensals | *Staphylococcus schleiferi* |
| Coagulase-negative staphylococci or common commensals | *Staphylococcus simulans* |
| Coagulase-negative staphylococci or common commensals | Staphylococcus species, coagulase-negative, unspeciated |
| Coagulase-negative staphylococci or common commensals | *Staphylococcus warneri* |
| Coagulase-negative staphylococci or common commensals | Tetragenococcus species |
| Enterococci | *Enterococcus avium* |
| Enterococci | *Enterococcus casseliflavus* |
| Enterococci | *Enterococcus cecorum* |
| Enterococci | *Enterococcus durans* |
| Enterococci | *Enterococcus faecalis* |
| Enterococci | *Enterococcus faecium* |
| Enterococci | *Enterococcus gallinarum* |
| Enterococci | *Enterococcus hirae* |
| Fungi | *Candida albicans* |
| Fungi | *Candida auris* |
| Fungi | *Candida dubliniensis* |
| Fungi | *Candida glabrata* |
| Fungi | *Candida guilliermondii* |
| Fungi | *Candida kefyr* |
| Fungi | *Candida krusei* |
| Fungi | *Candida lusitaniae* |
| Fungi | *Candida metapsilosis* |
| Fungi | *Candida orthopsilosis* |
| Fungi | *Candida parapsilosis* |
| Fungi | *Candida pelliculosa* |
| Fungi | Candida species |
| Fungi | *Candida tropicalis* |
| Fungi | Cryptococcus species |
| Fungi | *Cryptococcus neoformans* |
| Fungi | Fusarium species |
| Fungi | *Kodamaea ohmeri* |
| Fungi | *Lomentospora prolificans* |
| Fungi | *Rhodotorula musilaginosa* |
| Gram-negative rods | Achromobacter species |
| Gram-negative rods | *Acidovorax delafieldii* |
| Gram-negative rods | *Acinetobacter baumannii complex* |
| Gram-negative rods | *Acinetobacter lwoffii* |
| Gram-negative rods | Acinetobacter species |
| Gram-negative rods | *Acinetobacter ursingii* |
| Gram-negative rods | *Aeromonas sobria* |
| Gram-negative rods | Aeromonas species |
| Gram-negative rods | *Aggregatibacter aphrophilus* |
| Gram-negative rods | Alcaligenes species |
| Gram-negative rods | Bordetella species |
| Gram-negative rods | *Bordetella trematum* |
| Gram-negative rods | *Burkholderia cepacia complex* |
| Gram-negative rods | *Campylobacter jejuni* |
| Gram-negative rods | Capnocytophaga species |
| Gram-negative rods | *Cardiobacterium hominis* |
| Gram-negative rods | *Chryseobacterium indologenes* |
| Gram-negative rods | Chryseobacterium species |
| Gram-negative rods | *Citrobacter amalonaticus* |
| Gram-negative rods | *Citrobacter farmer* |
| Gram-negative rods | *Citrobacter freundii complex* |
| Gram-negative rods | *Citrobacter koseri* |
| Gram-negative rods | *Delftia acidovorans* |
| Gram-negative rods | *Eikenella corrodens* |
| Gram-negative rods | *Enterobacter cloacae complex* |
| Gram-negative rods | *Enterobacter hormaechei* |
| Gram-negative rods | *Escherichia coli* |
| Gram-negative rods | *Haemophilus influenzae* |
| Gram-negative rods | *Haemophilus parainfluenzae* |
| Gram-negative rods | *Hafnia alvei* |
| Gram-negative rods | Hafnia species |
| Gram-negative rods | *Klebsiella aerogenes* |
| Gram-negative rods | *Klebsiella oxytoca* |
| Gram-negative rods | *Klebsiella pneumoniae* |
| Gram-negative rods | *Klebsiella variicola* |
| Gram-negative rods | Kluyvera species |
| Gram-negative rods | *Leclercia adecarboxylata* |
| Gram-negative rods | *Listeria monocytogenes* |
| Gram-negative rods | Methylobacterium species |
| Gram-negative rods | *Moraxella canis* |
| Gram-negative rods | *Moraxella nonliquefaciens* |
| Gram-negative rods | *Moraxella osloensis* |
| Gram-negative rods | Moraxella species |
| Gram-negative rods | *Morganella morganii* |
| Gram-negative rods | Myroides species |
| Gram-negative rods | *Neisseria meningitidis* |
| Gram-negative rods | *Pantoea agglomerans* |
| Gram-negative rods | Pantoea species |
| Gram-negative rods | *Pasteurella multocida* |
| Gram-negative rods | *Pluralibacter gergoviae* |
| Gram-negative rods | *Proteus mirabilis* |
| Gram-negative rods | *Proteus penneri* |
| Gram-negative rods | *Proteus vulgaris* |
| Gram-negative rods | *Providencia rettgeri* |
| Gram-negative rods | Providencia species |
| Gram-negative rods | *Providencia stuartii* |
| Gram-negative rods | *Pseudescherichia vulneris* |
| Gram-negative rods | *Pseudomonas aeruginosa* |
| Gram-negative rods | *Pseudomonas fluorescens* |
| Gram-negative rods | *Pseudomonas oryzihabitans* |
| Gram-negative rods | *Pseudomonas putida* |
| Gram-negative rods | Pseudomonas species, not aeruginosa |
| Gram-negative rods | *Pseudomonas stutzeri* |
| Gram-negative rods | *Psychrobacter phenylpyruvicus* |
| Gram-negative rods | Rahnella species |
| Gram-negative rods | *Raoultella ornithinolytica* |
| Gram-negative rods | *Raoultella planticola* |
| Gram-negative rods | Raoultella species |
| Gram-negative rods | *Rhizobium* *radiobacter* |
| Gram-negative rods | Roseomonas species |
| Gram-negative rods | Salmonella species |
| Gram-negative rods | *Serratia liquefaciens* |
| Gram-negative rods | *Serratia marcescens* |
| Gram-negative rods | Shigella species |
| Gram-negative rods | *Sphingomonas paucimobilis* |
| Gram-negative rods | *Stenotrophomonas maltophilia* |
| Gram-negative rods | *Yersinia enterocolitica* |
| Rare organisms | *Arcanobacterium haemolyticum* |
| Rare organisms | *Bacillus cereus* |
| Rare organisms | Cruoricaptor species |
| Rare organisms | *Gardnerella vaginalis* |
| Rare organisms | Gordonia species |
| Rare organisms | *Mycobacterium abscessus complex* |
| Rare organisms | *Mycobacterium avium-intracellular complex* |
| Rare organisms | *Mycobacterium chelonae* |
| Rare organisms | *Mycobacterium fortuitum* group |
| Rare organisms | *Mycobacterium mucogenicum* |
| Rare organisms | Mycobacterium species |
| Rare organisms | *Neisseria cinerea* |
| Rare organisms | Neisseria species |
| Rare organisms | Tsukamurella species |
| *Staphylococcus aureus* or *lugdunensis* | *Staphylococcus aureus*, methicillin-resistant |
| *Staphylococcus aureus* or *lugdunensis* | *Staphylococcus aureus*, methicillin-susceptible |
| *Staphylococcus aureus* or *lugdunensis* | *Staphylococcus lugdunensis* |
| Streptococci | *Abiotrophia defectiva* |
| Streptococci | *Aerococcus urinae* |
| Streptococci | *Facklamia hominis* |
| Streptococci | *Gemella haemolysans* |
| Streptococci | *Gemella morbillorum* |
| Streptococci | Gemella species |
| Streptococci | Globicatella species |
| Streptococci | *Granulicatella adiacens* |
| Streptococci | Lactococcus species |
| Streptococci | *Streptococcus agalactiae* |
| Streptococci | *Streptococcus anginosus* |
| Streptococci | *Streptococcus constellatus* |
| Streptococci | *Streptococcus cristatus* |
| Streptococci | *Streptococcus dysgalactiae* |
| Streptococci | *Streptococcus gallolyticus* |
| Streptococci | *Streptococcus gordonii* |
| Streptococci | *Streptococcus infantarius* |
| Streptococci | *Streptococcus iniae* |
| Streptococci | *Streptococcus intermedius* |
| Streptococci | *Streptococcus mitis/oralis* |
| Streptococci | *Streptococcus mutans* |
| Streptococci | *Streptococcus parasanguinis* |
| Streptococci | *Streptococcus pneumoniae* |
| Streptococci | *Streptococcus pyogenes* |
| Streptococci | *Streptococcus salivarius* |
| Streptococci | *Streptococcus sanguinis* |
| Streptococci | Streptococcus species |
| Streptococci | Streptococcus species, β-hemolytic, group C |
| Streptococci | Streptococcus species, β-hemolytic, group G |
| Streptococci | *Streptococcus viridans* |

# **Supplementary Table 2.** Baseline Sociodemographic and Clinical Characteristics of Bloodstream Infection Episodes Aggregated by Time Period.

| **Characteristic, No. (%)** | **Pre-restriction**  **(N = 2 987)** | **Restriction**  **(N = 1 076)** | **Post-restriction**  **(N = 1 328)** | **Overall**  **(N = 5 391)** |
| --- | --- | --- | --- | --- |
| **Female sex** | 1 299 (43.5) | 470 (43.7) | 581 (43.8) | 2 350 (43.6) |
| **Age, years** |  | | | |
| <25 | 57 (1.9) | 25 (2.3) | 20 (1.5) | 102 (1.9) |
| 25–34 | 226 (7.6) | 89 (8.3) | 93 (7.0) | 408 (7.6) |
| 35–44 | 354 (11.9) | 118 (11.0) | 144 (10.8) | 616 (11.4) |
| 45–54 | 375 (12.6) | 130 (12.1) | 151 (11.4) | 656 (12.2) |
| 55–64 | 606 (20.3) | 281 (26.1) | 290 (21.8) | 1 177 (21.8) |
| 65–74 | 789 (26.4) | 239 (22.2) | 356 (26.8) | 1 384 (25.7) |
| 75–84 | 446 (14.9) | 147 (13.7) | 207 (15.6) | 800 (14.8) |
| ≥85 | 134 (4.5) | 47 (4.4) | 67 (5.0) | 248 (4.6) |
| **Race** |  | | | |
| Asian | 93 (3.1) | 35 (3.3) | 47 (3.5) | 175 (3.2) |
| Black | 1 391 (46.6) | 523 (48.6) | 636 (47.9) | 2 550 (47.3) |
| Other | 115 (3.9) | 35 (3.3) | 59 (4.4) | 209 (3.9) |
| Unknown | 141 (4.7) | 54 (5.0) | 74 (5.6) | 269 (5.0) |
| White | 1 247 (41.7) | 429 (39.9) | 512 (38.6) | 2 188 (40.6) |
| **Ethnicity** |  | | | |
| Hispanic/Latino | 89 (3.0) | 33 (3.1) | 35 (2.6) | 157 (2.9) |
| Non-Hispanic/Latino | 2 847 (95.3) | 1 020 (94.8) | 1 255 (94.5) | 5 122 (95.0) |
| Unknown | 51 (1.7) | 23 (2.1) | 38 (2.9) | 112 (2.1) |
| **Insurance** |  | | | |
| Private or commercial | 728 (24.4) | 283 (26.3) | 350 (26.4) | 1 361 (25.2) |
| Public, managed | 1 384 (46.3) | 505 (46.9) | 603 (45.4) | 2 492 (46.2) |
| Public, unmanaged or uninsured/self-pay | 875 (29.3) | 288 (26.8) | 375 (28.2) | 1 538 (28.5) |
| **Elixhauser Comorbidity Index** |  | | | |
| <0 | 89 (3.0) | 26 (2.4) | 21 (1.6) | 136 (2.5) |
| 0–9 | 473 (15.8) | 140 (13.0) | 237 (17.8) | 850 (15.8) |
| 10–19 | 605 (20.3) | 200 (18.6) | 236 (17.8) | 1 041 (19.3) |
| 20–29 | 589 (19.7) | 213 (19.8) | 293 (22.1) | 1 095 (20.3) |
| ≥30 | 1 231 (41.2) | 497 (46.2) | 541 (40.7) | 2 269 (42.1) |
| **Hospital** |  | | | |
| HUP | 1 887 (63.2) | 714 (66.4) | 854 (64.3) | 3 455 (64.1) |
| PPMC | 1 100 (36.8) | 362 (33.6) | 474 (35.7) | 1 936 (35.9) |

The pre-restriction, restriction, and post-restriction periods were June 26, 2023–June 25, 2024, June 26, 2024–December 23, 2024, and December 24, 2024–June 25, 2025, respectively. Data shown are the number of bloodstream infectious episodes having the indicated characteristic, with percentage in parentheses reflecting this as the numerator and size of the overall group as the denominator (shown in the headings).

Abbreviations: HUP, the Hospital of the University of Pennsylvania; PPMC, Penn Presbyterian Medical Center.

# **Supplementary Table 3.** Frequency of Individual Organisms Categorized as Anaerobes in Bloodstream Infection Episodes Aggregated by Time Period.

| **Anaerobes, No. (%)** | **Pre-restriction**  **(N = 132)** | **Restriction**  **(N = 34)** | **Post-restriction**  **(N = 61)** | **Overall**  **(N = 227)** |
| --- | --- | --- | --- | --- |
| *Bacteroides fragilis* | 25 (18.9) | 5 (14.7) | 10 (16.4) | 40 (17.6) |
| *Fusobacterium nucleatum* | 10 (7.6) | 4 (11.8) | 6 (9.8) | 20 (8.8) |
| Gram negative bacilli, anaerobic | 9 (6.8) | 3 (8.8) | 7 (11.5) | 19 (8.4) |
| *Bacteroides thetaiotaomicron* | 4 (3.0) | 3 (8.8) | 4 (6.6) | 11 (4.8) |
| Peptostreptococcus species | 4 (3.0) | 3 (8.8) | 3 (4.9) | 10 (4.4) |
| *Finegoldia magna* | 5 (3.8) | 3 (8.8) | 1 (1.6) | 9 (4.0) |
| *Parvimonas micra* | 7 (5.3) | 0 (0.0) | 2 (3.3) | 9 (4.0) |
| *Clostridium perfringens* | 7 (5.3) | 0 (0.0) | 1 (1.6) | 8 (3.5) |
| Gram positive cocci, anaerobic | 5 (3.8) | 0 (0.0) | 3 (4.9) | 8 (3.5) |
| Gram positive bacilli, anaerobic | 5 (3.8) | 0 (0.0) | 2 (3.3) | 7 (3.1) |
| Actinomyces species | 6 (4.5) | 0 (0.0) | 0 (0.0) | 6 (2.6) |
| *Bacteroides ovatus/xylanisolvens* | 0 (0.0) | 5 (14.7) | 1 (1.6) | 6 (2.6) |
| Bacteroides species | 2 (1.5) | 1 (2.9) | 2 (3.3) | 5 (2.2) |
| *Clostridium ramosum* | 3 (2.3) | 1 (2.9) | 1 (1.6) | 5 (2.2) |
| *Clostridium septicum* | 3 (2.3) | 0 (0.0) | 2 (3.3) | 5 (2.2) |
| *Prevotella bivia* | 4 (3.0) | 1 (2.9) | 0 (0.0) | 5 (2.2) |
| *Clostridium sporogenes* | 3 (2.3) | 0 (0.0) | 1 (1.6) | 4 (1.8) |
| *Bacteroides vulgatus* | 3 (2.3) | 0 (0.0) | 0 (0.0) | 3 (1.3) |
| Bifidobacterium species | 1 (0.8) | 1 (2.9) | 1 (1.6) | 3 (1.3) |
| Eggerthella species | 2 (1.5) | 1 (2.9) | 0 (0.0) | 3 (1.3) |
| Prevotella species | 2 (1.5) | 0 (0.0) | 1 (1.6) | 3 (1.3) |
| Veillonella species | 2 (1.5) | 0 (0.0) | 1 (1.6) | 3 (1.3) |
| *Actinomyces neuii* | 2 (1.5) | 0 (0.0) | 0 (0.0) | 2 (0.9) |
| *Clostridium tertium* | 2 (1.5) | 0 (0.0) | 0 (0.0) | 2 (0.9) |
| *Bacteroides eggerthii* | 1 (0.8) | 0 (0.0) | 0 (0.0) | 1 (0.4) |
| *Bacteroides pyogenes* | 0 (0.0) | 0 (0.0) | 1 (1.6) | 1 (0.4) |
| *Bacteroides uniformis* | 0 (0.0) | 0 (0.0) | 1 (1.6) | 1 (0.4) |
| *Bacteroides vulgatus/thetaiotaomicron* | 1 (0.8) | 0 (0.0) | 0 (0.0) | 1 (0.4) |
| *Clostridium clostridioforme* | 0 (0.0) | 0 (0.0) | 1 (1.6) | 1 (0.4) |
| *Clostridium innocuum* | 1 (0.8) | 0 (0.0) | 0 (0.0) | 1 (0.4) |
| Clostridium species | 0 (0.0) | 0 (0.0) | 1 (1.6) | 1 (0.4) |
| *Fusobacterium periodonticum* | 0 (0.0) | 1 (2.9) | 0 (0.0) | 1 (0.4) |
| Gram variable bacilli, anaerobic | 1 (0.8) | 0 (0.0) | 0 (0.0) | 1 (0.4) |
| Lancefieldella species | 0 (0.0) | 0 (0.0) | 1 (1.6) | 1 (0.4) |
| *Paeniclostridium sordellii* | 0 (0.0) | 1 (2.9) | 0 (0.0) | 1 (0.4) |
| Peptoniphilus species | 0 (0.0) | 0 (0.0) | 1 (1.6) | 1 (0.4) |
| *Porphyromonas asaccharolytica* | 1 (0.8) | 0 (0.0) | 0 (0.0) | 1 (0.4) |
| *Prevotella buccae* | 1 (0.8) | 0 (0.0) | 0 (0.0) | 1 (0.4) |
| *Prevotella melaninogenica* | 0 (0.0) | 0 (0.0) | 1 (1.6) | 1 (0.4) |
| Tissierella species | 1 (0.8) | 0 (0.0) | 0 (0.0) | 1 (0.4) |
| *Veillonella dispar* | 1 (0.8) | 0 (0.0) | 0 (0.0) | 1 (0.4) |
| *Weissella confusa* | 1 (0.8) | 0 (0.0) | 0 (0.0) | 1 (0.4) |
| Weissella species | 1 (0.8) | 0 (0.0) | 0 (0.0) | 1 (0.4) |
| *Arcanobacterium haemolyticum* \| *Peptostreptococcus species* | 1 (0.8) | 0 (0.0) | 0 (0.0) | 1 (0.4) |
| *Bacteroides caccae* \| *Bacteroides fragilis* \| *Clostridium ramosum* | 0 (0.0) | 0 (0.0) | 1 (1.6) | 1 (0.4) |
| *Bacteroides fragilis* \| *Finegoldia magna* | 1 (0.8) | 0 (0.0) | 0 (0.0) | 1 (0.4) |
| *Bacteroides fragilis* \| Fusobacterium species \| *Peptoniphilus asaccharolyticus* | 1 (0.8) | 0 (0.0) | 0 (0.0) | 1 (0.4) |
| *Bacteroides fragilis* \| *Prevotella intermedia* | 0 (0.0) | 0 (0.0) | 1 (1.6) | 1 (0.4) |
| *Bacteroides fragilis* \| Prevotella species | 0 (0.0) | 0 (0.0) | 1 (1.6) | 1 (0.4) |
| *Bacteroides ovatus/xylanisolvens/vulgatus* \| *Bacteroides vulgatus* \| *Finegoldia magna* | 1 (0.8) | 0 (0.0) | 0 (0.0) | 1 (0.4) |
| *Bacteroides thetaiotaomicron* \| *Peptoniphilus asaccharolyticus* | 1 (0.8) | 0 (0.0) | 0 (0.0) | 1 (0.4) |
| *Bacteroides uniformis* \| *Bacteroides vulgatus* | 0 (0.0) | 0 (0.0) | 1 (1.6) | 1 (0.4) |
| *Bacteroides vulgatus* \| Eggerthella species | 1 (0.8) | 0 (0.0) | 0 (0.0) | 1 (0.4) |
| *Bacteroides vulgatus/thetaiotaomicron* | 1 (0.8) | 0 (0.0) | 0 (0.0) | 1 (0.4) |
| *Clostridium cadaveris* \| *Clostridium sporogenes* | 1 (0.8) | 0 (0.0) | 0 (0.0) | 1 (0.4) |
| *Parvimonas micra* \| *Prevotella buccae* | 0 (0.0) | 1 (2.9) | 0 (0.0) | 1 (0.4) |
| *Prevotella melaninogenica* \| *Schaalia odontolyticus* | 0 (0.0) | 0 (0.0) | 1 (1.6) | 1 (0.4) |

The pre-restriction, restriction, and post-restriction periods were June 26, 2023–June 25, 2024, June 26, 2024–December 23, 2024, and December 24, 2024–June 25, 2025, respectively. Data shown are the number of bloodstream infectious episodes with the listed organism(s), with percentage in parentheses reflecting this as the numerator and size of the overall group as the denominator (shown in the headings). Single-organism episodes are listed first in order from most to least frequent, followed by multi-organism episodes in order from most to least frequent.

# **Supplementary Table 4.** Frequency of Individual Organisms Categorized as Enterococci in Bloodstream Infection Episodes Aggregated by Time Period.

| **Enterococci, No. (%)** | **Pre-restriction**  **(N = 156)** | **Restriction**  **(N = 79)** | **Post-restriction**  **(N = 89)** | **Overall**  **(N = 324)** |
| --- | --- | --- | --- | --- |
| *Enterococcus faecalis* | 95 (60.9) | 50 (63.3) | 50 (56.2) | 195 (60.2) |
| *Enterococcus faecium* | 56 (35.9) | 25 (31.6) | 34 (38.2) | 115 (35.5) |
| *Enterococcus gallinarum* | 0 (0.0) | 2 (2.5) | 1 (1.1) | 3 (0.9) |
| *Enterococcus hirae* | 1 (0.6) | 1 (1.3) | 0 (0.0) | 2 (0.6) |
| *Enterococcus avium* | 0 (0.0) | 1 (1.3) | 0 (0.0) | 1 (0.3) |
| *Enterococcus casseliflavus* | 0 (0.0) | 0 (0.0) | 1 (1.1) | 1 (0.3) |
| *Enterococcus cecorum* | 1 (0.6) | 0 (0.0) | 0 (0.0) | 1 (0.3) |
| *Enterococcus durans* | 1 (0.6) | 0 (0.0) | 0 (0.0) | 1 (0.3) |
| *Enterococcus faecalis* \| *Enterococcus faecium* | 1 (0.6) | 0 (0.0) | 3 (3.4) | 4 (1.2) |
| *Bacillus cereus* \| *Enterococcus faecalis* | 1 (0.6) | 0 (0.0) | 0 (0.0) | 1 (0.3) |

The pre-restriction, restriction, and post-restriction periods were June 26, 2023–June 25, 2024, June 26, 2024–December 23, 2024, and December 24, 2024–June 25, 2025, respectively. Data shown are the number of bloodstream infectious episodes with the listed organism(s), with percentage in parentheses reflecting this as the numerator and size of the overall group as the denominator (shown in the headings). Single-organism episodes are listed first in order from most to least frequent, followed by multi-organism episodes in order from most to least frequent.

# **Supplementary Table 5.** Frequency of Individual Organisms Categorized as Fungi in Bloodstream Infection Episodes Aggregated by Time Period.

| **Fungi, No. (%)** | **Pre-restriction**  **(N = 118)** | **Restriction**  **(N = 62)** | **Post-restriction**  **(N = 53)** | **Overall**  **(N = 233)** |
| --- | --- | --- | --- | --- |
| *Candida albicans* | 41 (34.7) | 20 (32.3) | 14 (26.4) | 75 (32.2) |
| *Candida glabrata* | 24 (20.3) | 13 (21.0) | 19 (35.8) | 56 (24.0) |
| *Candida parapsilosis* | 30 (25.4) | 17 (27.4) | 4 (7.5) | 51 (21.9) |
| *Candida tropicalis* | 6 (5.1) | 1 (1.6) | 5 (9.4) | 12 (5.2) |
| *Candida auris* | 5 (4.2) | 0 (0.0) | 5 (9.4) | 10 (4.3) |
| *Candida orthopsilosis* | 1 (0.8) | 1 (1.6) | 2 (3.8) | 4 (1.7) |
| *Candida krusei* | 1 (0.8) | 1 (1.6) | 1 (1.9) | 3 (1.3) |
| *Candida lusitaniae* | 3 (2.5) | 0 (0.0) | 0 (0.0) | 3 (1.3) |
| *Cryptococcus neoformans* | 2 (1.7) | 0 (0.0) | 1 (1.9) | 3 (1.3) |
| Fusarium species | 0 (0.0) | 1 (1.6) | 2 (3.8) | 3 (1.3) |
| *Rhodotorula musilaginosa* | 2 (1.7) | 1 (1.6) | 0 (0.0) | 3 (1.3) |
| *Candida dubliniensis* | 0 (0.0) | 1 (1.6) | 0 (0.0) | 1 (0.4) |
| *Candida guilliermondii* | 0 (0.0) | 1 (1.6) | 0 (0.0) | 1 (0.4) |
| *Candida metapsilosis* | 0 (0.0) | 1 (1.6) | 0 (0.0) | 1 (0.4) |
| Candida species | 0 (0.0) | 1 (1.6) | 0 (0.0) | 1 (0.4) |
| Cryptococcus | 0 (0.0) | 1 (1.6) | 0 (0.0) | 1 (0.4) |
| *Kodamaea ohmeri* | 1 (0.8) | 0 (0.0) | 0 (0.0) | 1 (0.4) |
| *Lomentospora prolificans* | 1 (0.8) | 0 (0.0) | 0 (0.0) | 1 (0.4) |
| *Candida albicans* \| *Candida glabrata* | 1 (0.8) | 1 (1.6) | 0 (0.0) | 2 (0.9) |
| *Candida glabrata* \| *Candida tropicalis* | 0 (0.0) | 1 (1.6) | 0 (0.0) | 1 (0.4) |

The pre-restriction, restriction, and post-restriction periods were June 26, 2023–June 25, 2024, June 26, 2024–December 23, 2024, and December 24, 2024–June 25, 2025, respectively. Data shown are the number of bloodstream infectious episodes with the listed organism(s), with percentage in parentheses reflecting this as the numerator and size of the overall group as the denominator (shown in the headings). Single-organism episodes are listed first in order from most to least frequent, followed by multi-organism episodes in order from most to least frequent.

# **Supplementary Table 6.** Frequency of Individual Organisms Categorized as Gram-Negative Rods in Bloodstream Infection Episodes Aggregated by Time Period.

| **Gram-negative rods, No. (%)** | **Pre-restriction**  **(N = 761)** | **Restriction**  **(N = 323)** | **Post-restriction**  **(N = 366)** | **Overall**  **(N = 1 450)** |
| --- | --- | --- | --- | --- |
| *Escherichia coli* | 253 (33.2) | 115 (35.6) | 127 (34.7) | 495 (34.1) |
| *Klebsiella pneumoniae* | 134 (17.6) | 59 (18.3) | 77 (21.0) | 270 (18.6) |
| *Pseudomonas aeruginosa* | 76 (10.0) | 37 (11.5) | 27 (7.4) | 140 (9.7) |
| *Enterobacter cloacae* complex | 49 (6.4) | 28 (8.7) | 23 (6.3) | 100 (6.9) |
| *Proteus mirabilis* | 26 (3.4) | 18 (5.6) | 13 (3.6) | 57 (3.9) |
| *Serratia marcescens* | 25 (3.3) | 7 (2.2) | 9 (2.5) | 41 (2.8) |
| *Klebsiella oxytoca* | 16 (2.1) | 3 (0.9) | 12 (3.3) | 31 (2.1) |
| *Klebsiella aerogenes* | 10 (1.3) | 5 (1.5) | 8 (2.2) | 23 (1.6) |
| *Haemophilus influenzae* | 12 (1.6) | 3 (0.9) | 5 (1.4) | 20 (1.4) |
| *Citrobacter freundii* complex | 12 (1.6) | 4 (1.2) | 0 (0.0) | 16 (1.1) |
| *Acinetobacter baumannii* complex | 11 (1.4) | 0 (0.0) | 3 (0.8) | 14 (1.0) |
| *Stenotrophomonas maltophilia* | 6 (0.8) | 2 (0.6) | 6 (1.6) | 14 (1.0) |
| *Morganella morganii* | 6 (0.8) | 4 (1.2) | 3 (0.8) | 13 (0.9) |
| Salmonella species | 6 (0.8) | 5 (1.5) | 2 (0.5) | 13 (0.9) |
| Gram negative bacilli | 7 (0.9) | 0 (0.0) | 2 (0.5) | 9 (0.6) |
| *Klebsiella pneumoniae/variicola* | 3 (0.4) | 2 (0.6) | 3 (0.8) | 8 (0.6) |
| Shigella species | 5 (0.7) | 2 (0.6) | 1 (0.3) | 8 (0.6) |
| Acinetobacter species | 3 (0.4) | 1 (0.3) | 3 (0.8) | 7 (0.5) |
| Moraxella species | 4 (0.5) | 0 (0.0) | 2 (0.5) | 6 (0.4) |
| *Citrobacter koseri* | 0 (0.0) | 3 (0.9) | 2 (0.5) | 5 (0.3) |
| *Listeria monocytogenes* | 3 (0.4) | 1 (0.3) | 1 (0.3) | 5 (0.3) |
| *Providencia rettgeri* | 2 (0.3) | 2 (0.6) | 1 (0.3) | 5 (0.3) |
| *Raoultella ornithinolytica* | 2 (0.3) | 2 (0.6) | 1 (0.3) | 5 (0.3) |
| Achromobacter species | 3 (0.4) | 1 (0.3) | 0 (0.0) | 4 (0.3) |
| Aeromonas species | 2 (0.3) | 1 (0.3) | 0 (0.0) | 3 (0.2) |
| Capnocytophaga species | 3 (0.4) | 0 (0.0) | 0 (0.0) | 3 (0.2) |
| *Cardiobacterium hominis* | 1 (0.1) | 0 (0.0) | 2 (0.5) | 3 (0.2) |
| *Hafnia alvei* | 1 (0.1) | 1 (0.3) | 1 (0.3) | 3 (0.2) |
| *Pantoea agglomerans* | 0 (0.0) | 1 (0.3) | 2 (0.5) | 3 (0.2) |
| *Acinetobacter lwoffii* | 2 (0.3) | 0 (0.0) | 0 (0.0) | 2 (0.1) |
| Bordetella species | 1 (0.1) | 1 (0.3) | 0 (0.0) | 2 (0.1) |
| *Burkholderia cepacia* complex | 2 (0.3) | 0 (0.0) | 0 (0.0) | 2 (0.1) |
| Pantoea species | 2 (0.3) | 0 (0.0) | 0 (0.0) | 2 (0.1) |
| *Pluralibacter gergoviae* | 2 (0.3) | 0 (0.0) | 0 (0.0) | 2 (0.1) |
| *Pseudomonas oryzihabitans* | 1 (0.1) | 0 (0.0) | 1 (0.3) | 2 (0.1) |
| Pseudomonas species, not aeruginosa | 1 (0.1) | 1 (0.3) | 0 (0.0) | 2 (0.1) |
| *Pseudomonas stutzeri* | 0 (0.0) | 0 (0.0) | 2 (0.5) | 2 (0.1) |
| *Serratia liquefaciens* | 0 (0.0) | 1 (0.3) | 1 (0.3) | 2 (0.1) |
| *Sphingomonas paucimobilis* | 2 (0.3) | 0 (0.0) | 0 (0.0) | 2 (0.1) |
| *Acidovorax delafieldii* | 1 (0.1) | 0 (0.0) | 0 (0.0) | 1 (0.1) |
| *Aggregatibacter aphrophilus* | 1 (0.1) | 0 (0.0) | 0 (0.0) | 1 (0.1) |
| *Campylobacter jejuni* | 1 (0.1) | 0 (0.0) | 0 (0.0) | 1 (0.1) |
| Chryseobacterium species | 1 (0.1) | 0 (0.0) | 0 (0.0) | 1 (0.1) |
| *Citrobacter amalonaticus* | 0 (0.0) | 0 (0.0) | 1 (0.3) | 1 (0.1) |
| *Delftia acidovorans* | 1 (0.1) | 0 (0.0) | 0 (0.0) | 1 (0.1) |
| *Haemophilus parainfluenzae* | 1 (0.1) | 0 (0.0) | 0 (0.0) | 1 (0.1) |
| *Klebsiella variicola* | 1 (0.1) | 0 (0.0) | 0 (0.0) | 1 (0.1) |
| *Moraxella canis* | 1 (0.1) | 0 (0.0) | 0 (0.0) | 1 (0.1) |
| *Moraxella nonliquefaciens* | 1 (0.1) | 0 (0.0) | 0 (0.0) | 1 (0.1) |
| *Moraxella osloensis* | 0 (0.0) | 0 (0.0) | 1 (0.3) | 1 (0.1) |
| *Neisseria meningitidis* | 1 (0.1) | 0 (0.0) | 0 (0.0) | 1 (0.1) |
| *Proteus penneri* | 1 (0.1) | 0 (0.0) | 0 (0.0) | 1 (0.1) |
| *Proteus vulgaris* | 1 (0.1) | 0 (0.0) | 0 (0.0) | 1 (0.1) |
| Providencia species | 0 (0.0) | 1 (0.3) | 0 (0.0) | 1 (0.1) |
| *Providencia stuartii* | 0 (0.0) | 1 (0.3) | 0 (0.0) | 1 (0.1) |
| *Pseudescherichia vulneris* | 1 (0.1) | 0 (0.0) | 0 (0.0) | 1 (0.1) |
| *Pseudomonas putida* | 1 (0.1) | 0 (0.0) | 0 (0.0) | 1 (0.1) |
| *Raoultella planticola* | 0 (0.0) | 1 (0.3) | 0 (0.0) | 1 (0.1) |
| *Yersinia enterocolitica* | 0 (0.0) | 0 (0.0) | 1 (0.3) | 1 (0.1) |
| *Escherichia coli* \| *Klebsiella pneumoniae* | 6 (0.8) | 5 (1.5) | 7 (1.9) | 18 (1.2) |
| *Escherichia coli* \| *Klebsiella oxytoca* | 4 (0.5) | 0 (0.0) | 0 (0.0) | 4 (0.3) |
| *Escherichia coli* \| *Pseudomonas aeruginosa* | 4 (0.5) | 0 (0.0) | 0 (0.0) | 4 (0.3) |
| *Klebsiella pneumoniae* \| *Pseudomonas aeruginosa* | 2 (0.3) | 1 (0.3) | 1 (0.3) | 4 (0.3) |
| *Acinetobacter baumannii* complex \| *Enterobacter cloacae* complex | 1 (0.1) | 0 (0.0) | 1 (0.3) | 2 (0.1) |
| *Enterobacter cloacae* complex \| *Klebsiella pneumoniae* | 1 (0.1) | 0 (0.0) | 1 (0.3) | 2 (0.1) |
| *Enterobacter cloacae* complex \| *Pseudomonas aeruginosa* | 1 (0.1) | 1 (0.3) | 0 (0.0) | 2 (0.1) |
| *Escherichia coli* \| *Proteus mirabilis* | 1 (0.1) | 0 (0.0) | 1 (0.3) | 2 (0.1) |
| *Klebsiella pneumoniae* \| *Proteus mirabilis* | 1 (0.1) | 0 (0.0) | 1 (0.3) | 2 (0.1) |
| *Klebsiella pneumoniae* \| *Serratia marcescens* | 1 (0.1) | 0 (0.0) | 1 (0.3) | 2 (0.1) |
| *Morganella morganii* \| *Proteus mirabilis* | 1 (0.1) | 1 (0.3) | 0 (0.0) | 2 (0.1) |
| *Pseudomonas aeruginosa* \| *Serratia marcescens* | 2 (0.3) | 0 (0.0) | 0 (0.0) | 2 (0.1) |
| Achromobacter species \| *Proteus mirabilis* | 0 (0.0) | 0 (0.0) | 1 (0.3) | 1 (0.1) |
| *Acinetobacter baumannii* complex \| *Enterobacter cloacae* complex \| Myroides species | 0 (0.0) | 1 (0.3) | 0 (0.0) | 1 (0.1) |
| *Acinetobacter baumannii* complex \| *Leclercia adecarboxylata* | 0 (0.0) | 1 (0.3) | 0 (0.0) | 1 (0.1) |
| *Acinetobacter baumannii* complex \| Moraxella species \| *Sphingomonas paucimobilis* | 1 (0.1) | 0 (0.0) | 0 (0.0) | 1 (0.1) |
| Acinetobacter species \| *Chryseobacterium indologenes* *Stenotrophomonas maltophilia* | 1 (0.1) | 0 (0.0) | 0 (0.0) | 1 (0.1) |
| Acinetobacter species \| Rahnella species \| *Stenotrophomonas maltophilia* | 1 (0.1) | 0 (0.0) | 0 (0.0) | 1 (0.1) |
| Acinetobacter species \| *Rhizobium radiobacter* \| *Stenotrophomonas maltophilia* | 1 (0.1) | 0 (0.0) | 0 (0.0) | 1 (0.1) |
| *Acinetobacter ursingii* \| *Klebsiella oxytoca* | 0 (0.0) | 0 (0.0) | 1 (0.3) | 1 (0.1) |
| *Aeromonas sobria* \| *Escherichia coli* | 1 (0.1) | 0 (0.0) | 0 (0.0) | 1 (0.1) |
| Aeromonas species \| *Enterobacter cloacae* complex \| *Klebsiella pneumoniae* | 1 (0.1) | 0 (0.0) | 0 (0.0) | 1 (0.1) |
| Aeromonas species \| *Escherichia coli* \| *Klebsiella aerogenes* | 1 (0.1) | 0 (0.0) | 0 (0.0) | 1 (0.1) |
| Alcaligenes species \| Myroides species | 0 (0.0) | 0 (0.0) | 1 (0.3) | 1 (0.1) |
| Bordetella species \| *Escherichia coli* \| *Pseudomonas aeruginosa* | 0 (0.0) | 0 (0.0) | 1 (0.3) | 1 (0.1) |
| *Citrobacter freundii* complex \| *Klebsiella pneumoniae/variicola* | 1 (0.1) | 0 (0.0) | 0 (0.0) | 1 (0.1) |
| *Citrobacter koseri* \| *Klebsiella oxytoca* | 1 (0.1) | 0 (0.0) | 0 (0.0) | 1 (0.1) |
| *Citrobacter koseri* \| *Klebsiella pneumoniae* | 0 (0.0) | 0 (0.0) | 1 (0.3) | 1 (0.1) |
| *Enterobacter cloacae* complex \| *Enterobacter hormaechei* | 1 (0.1) | 0 (0.0) | 0 (0.0) | 1 (0.1) |
| *Enterobacter cloacae* complex \| *Klebsiella pneumoniae* \| *Serratia marcescens* | 1 (0.1) | 0 (0.0) | 0 (0.0) | 1 (0.1) |
| *Enterobacter cloacae* complex \| *Proteus mirabilis* | 0 (0.0) | 0 (0.0) | 1 (0.3) | 1 (0.1) |
| *Escherichia coli* \| *Haemophilus influenzae* | 1 (0.1) | 0 (0.0) | 0 (0.0) | 1 (0.1) |
| *Escherichia coli* \| *Klebsiella pneumoniae* \| *Proteus mirabilis* | 1 (0.1) | 0 (0.0) | 0 (0.0) | 1 (0.1) |
| *Escherichia coli* \| *Listeria monocytogenes* | 1 (0.1) | 0 (0.0) | 0 (0.0) | 1 (0.1) |
| *Escherichia coli* \| *Morganella morganii* | 0 (0.0) | 0 (0.0) | 1 (0.3) | 1 (0.1) |
| *Escherichia coli* \| *Providencia rettgeri* | 0 (0.0) | 0 (0.0) | 1 (0.3) | 1 (0.1) |
| *Escherichia coli* \| *Raoultella ornithinolytica* | 1 (0.1) | 0 (0.0) | 0 (0.0) | 1 (0.1) |
| *Escherichia coli* \| *Serratia marcescens* | 1 (0.1) | 0 (0.0) | 0 (0.0) | 1 (0.1) |
| Hafnia species \| *Raoultella ornithinolytica* | 1 (0.1) | 0 (0.0) | 0 (0.0) | 1 (0.1) |
| *Klebsiella aerogenes* \| *Klebsiella pneumoniae* | 1 (0.1) | 0 (0.0) | 0 (0.0) | 1 (0.1) |
| *Klebsiella aerogenes* \| *Proteus mirabilis* | 1 (0.1) | 0 (0.0) | 0 (0.0) | 1 (0.1) |
| *Klebsiella oxytoca* \| *Klebsiella pneumoniae* | 0 (0.0) | 0 (0.0) | 1 (0.3) | 1 (0.1) |
| *Klebsiella oxytoca* \| *Pantoea agglomerans* | 1 (0.1) | 0 (0.0) | 0 (0.0) | 1 (0.1) |
| *Klebsiella oxytoca* \| *Pseudomonas aeruginosa* | 1 (0.1) | 0 (0.0) | 0 (0.0) | 1 (0.1) |
| *Klebsiella pneumoniae* \| Paenibacillus species | 1 (0.1) | 0 (0.0) | 0 (0.0) | 1 (0.1) |
| *Klebsiella pneumoniae* \| *Proteus mirabilis* \| *Pseudomonas aeruginosa* | 0 (0.0) | 0 (0.0) | 1 (0.3) | 1 (0.1) |
| *Klebsiella pneumoniae* \| *Stenotrophomonas maltophilia* | 1 (0.1) | 0 (0.0) | 0 (0.0) | 1 (0.1) |
| *Klebsiella pneumoniae/variicola* \| *Proteus mirabilis* | 1 (0.1) | 0 (0.0) | 0 (0.0) | 1 (0.1) |
| *Pluralibacter gergoviae* \| *Serratia liquefaciens* | 1 (0.1) | 0 (0.0) | 0 (0.0) | 1 (0.1) |
| *Proteus mirabilis* \| *Pseudomonas aeruginosa* | 1 (0.1) | 0 (0.0) | 0 (0.0) | 1 (0.1) |
| *Pseudomonas aeruginosa* \| *Raoultella ornithinolytica* | 1 (0.1) | 0 (0.0) | 0 (0.0) | 1 (0.1) |
| *Raoultella planticola* \| *Serratia marcescens* | 1 (0.1) | 0 (0.0) | 0 (0.0) | 1 (0.1) |

The pre-restriction, restriction, and post-restriction periods were June 26, 2023–June 25, 2024, June 26, 2024–December 23, 2024, and December 24, 2024–June 25, 2025, respectively. Data shown are the number of bloodstream infectious episodes with the listed organism(s), with percentage in parentheses reflecting this as the numerator and size of the overall group as the denominator (shown in the headings). Single-organism episodes are listed first in order from most to least frequent, followed by multi-organism episodes in order from most to least frequent.

# **Supplementary Table 7.** Frequency of Individual Organisms Categorized as *Staphylococcus aureus* or *lugdunensis* in Bloodstream Infection Episodes Aggregated by Time Period.

| ***Staphylococcus aureus* or *lugdunensis*, No. (%)** | **Pre-restriction**  **(N = 336)** | **Restriction**  **(N = 159)** | **Post-restriction**  **(N = 158)** | **Overall**  **(N = 653)** |
| --- | --- | --- | --- | --- |
| *Staphylococcus aureus*, methicillin-susceptible | 177 (52.7) | 80 (50.3) | 84 (53.2) | 341 (52.2) |
| *Staphylococcus aureus*, methicillin-resistant | 147 (43.8) | 75 (47.2) | 71 (44.9) | 293 (44.9) |
| *Staphylococcus lugdunensis* | 10 (3.0) | 3 (1.9) | 3 (1.9) | 16 (2.5) |
| *Staphylococcus aureus*, methicillin-resistant \| *Staphylococcus aureus*, methicillin-susceptible | 2 (0.6) | 1 (0.6) | 0 (0.0) | 3 (0.5) |

The pre-restriction, restriction, and post-restriction periods were June 26, 2023–June 25, 2024, June 26, 2024–December 23, 2024, and December 24, 2024–June 25, 2025, respectively. Data shown are the number of bloodstream infectious episodes with the listed organism(s), with percentage in parentheses reflecting this as the numerator and size of the overall group as the denominator (shown in the headings). Single-organism episodes are listed first in order from most to least frequent, followed by multi-organism episodes in order from most to least frequent.

# **Supplementary Table 8.** Frequency of Individual Organisms Categorized as Streptococci in Bloodstream Infection Episodes Aggregated by Time Period.

| **Streptococci, No. (%)** | **Pre-restriction**  **(N = 301)** | **Restriction**  **(N = 82)** | **Post-restriction**  **(N = 123)** | **Overall**  **(N = 506)** |
| --- | --- | --- | --- | --- |
| *Streptococcus pyogenes* | 54 (17.9) | 15 (18.3) | 17 (13.8) | 86 (17.0) |
| *Streptococcus pneumoniae* | 37 (12.3) | 6 (7.3) | 21 (17.1) | 64 (12.6) |
| *Streptococcus mitis/oralis* | 38 (12.6) | 11 (13.4) | 13 (10.6) | 62 (12.3) |
| *Streptococcus agalactiae* | 33 (11.0) | 9 (11.0) | 10 (8.1) | 52 (10.3) |
| *Streptococcus dysgalactiae* | 28 (9.3) | 6 (7.3) | 5 (4.1) | 39 (7.7) |
| *Streptococcus anginosus* | 12 (4.0) | 7 (8.5) | 12 (9.8) | 31 (6.1) |
| *Streptococcus parasanguinis* | 15 (5.0) | 3 (3.7) | 2 (1.6) | 20 (4.0) |
| *Streptococcus constellatus* | 6 (2.0) | 5 (6.1) | 5 (4.1) | 16 (3.2) |
| *Granulicatella adiacens* | 3 (1.0) | 3 (3.7) | 7 (5.7) | 13 (2.6) |
| *Streptococcus salivarius* | 7 (2.3) | 3 (3.7) | 2 (1.6) | 12 (2.4) |
| *Aerococcus urinae* | 4 (1.3) | 3 (3.7) | 3 (2.4) | 10 (2.0) |
| *Streptococcus gordonii* | 5 (1.7) | 1 (1.2) | 3 (2.4) | 9 (1.8) |
| *Streptococcus intermedius* | 5 (1.7) | 0 (0.0) | 4 (3.3) | 9 (1.8) |
| *Streptococcus viridans* | 9 (3.0) | 0 (0.0) | 0 (0.0) | 9 (1.8) |
| *Streptococcus sanguinis* | 5 (1.7) | 1 (1.2) | 2 (1.6) | 8 (1.6) |
| *Streptococcus mutans* | 4 (1.3) | 1 (1.2) | 0 (0.0) | 5 (1.0) |
| *Aerococcus viridans* | 1 (0.3) | 0 (0.0) | 3 (2.4) | 4 (0.8) |
| *Gemella haemolysans* | 2 (0.7) | 1 (1.2) | 1 (0.8) | 4 (0.8) |
| *Streptococcus gallolyticus* | 2 (0.7) | 1 (1.2) | 1 (0.8) | 4 (0.8) |
| *Gemella morbillorum* | 2 (0.7) | 1 (1.2) | 0 (0.0) | 3 (0.6) |
| Globicatella species | 3 (1.0) | 0 (0.0) | 0 (0.0) | 3 (0.6) |
| *Streptococcus cristatus* | 2 (0.7) | 0 (0.0) | 1 (0.8) | 3 (0.6) |
| Streptococcus species | 2 (0.7) | 1 (1.2) | 0 (0.0) | 3 (0.6) |
| Streptococcus species, beta-hemolytic, group G | 2 (0.7) | 1 (1.2) | 0 (0.0) | 3 (0.6) |
| *Streptococcus infantarius* | 1 (0.3) | 0 (0.0) | 1 (0.8) | 2 (0.4) |
| *Abiotrophia defectiva* | 1 (0.3) | 0 (0.0) | 0 (0.0) | 1 (0.2) |
| Facklamia species | 0 (0.0) | 0 (0.0) | 1 (0.8) | 1 (0.2) |
| Gemella species | 1 (0.3) | 0 (0.0) | 0 (0.0) | 1 (0.2) |
| *Streptococcus iniae* | 1 (0.3) | 0 (0.0) | 0 (0.0) | 1 (0.2) |
| *Streptococcus anginosus* \| *Streptococcus constellatus* | 2 (0.7) | 0 (0.0) | 0 (0.0) | 2 (0.4) |
| *Streptococcus anginosus* \| *Streptococcus pyogenes* | 1 (0.3) | 0 (0.0) | 1 (0.8) | 2 (0.4) |
| *Streptococcus mitis/oralis* \| *Streptococcus salivarius* | 1 (0.3) | 0 (0.0) | 1 (0.8) | 2 (0.4) |
| *Streptococcus parasanguinis* \| *Streptococcus salivarius* | 2 (0.7) | 0 (0.0) | 0 (0.0) | 2 (0.4) |
| *Abiotrophia defectiva* \| *Streptococcus sanguinis* | 1 (0.3) | 0 (0.0) | 0 (0.0) | 1 (0.2) |
| *Abiotrophia defectiva* \| *Streptococcus viridans* | 1 (0.3) | 0 (0.0) | 0 (0.0) | 1 (0.2) |
| *Aerococcus urinae* \| *Streptococcus anginosus* | 0 (0.0) | 0 (0.0) | 1 (0.8) | 1 (0.2) |
| *Aerococcus viridans* \| Globicatella species | 1 (0.3) | 0 (0.0) | 0 (0.0) | 1 (0.2) |
| *Gemella haemolysans* \| *Granulicatella adiacens* \| *Streptococcus mitis/oralis* | 1 (0.3) | 0 (0.0) | 0 (0.0) | 1 (0.2) |
| *Gemella morbillorum* \| *Streptococcus anginosus* | 0 (0.0) | 1 (1.2) | 0 (0.0) | 1 (0.2) |
| *Gemella morbillorum* \| *Streptococcus mitis/oralis* \| *Streptococcus parasanguinis* | 1 (0.3) | 0 (0.0) | 0 (0.0) | 1 (0.2) |
| *Granulicatella adiacens* \| *Streptococcus mitis/oralis* \| *Streptococcus parasanguinis* | 0 (0.0) | 0 (0.0) | 1 (0.8) | 1 (0.2) |
| *Granulicatella adiacens* \| *Streptococcus mitis/oralis* \| *Streptococcus salivarius* | 0 (0.0) | 0 (0.0) | 1 (0.8) | 1 (0.2) |
| *Granulicatella adiacens* \| *Streptococcus salivarius* | 0 (0.0) | 1 (1.2) | 0 (0.0) | 1 (0.2) |
| Lactococcus species \| *Streptococcus mitis/oralis* \| *Streptococcus salivarius* | 0 (0.0) | 0 (0.0) | 1 (0.8) | 1 (0.2) |
| *Streptococcus anginosus* \| *Streptococcus dysgalactiae* \| *Streptococcus salivarius* | 0 (0.0) | 0 (0.0) | 1 (0.8) | 1 (0.2) |
| *Streptococcus anginosus* \| *Streptococcus parasanguinis* \| *Streptococcus salivarius* | 1 (0.3) | 0 (0.0) | 0 (0.0) | 1 (0.2) |
| *Streptococcus anginosus* \| *Streptococcus sanguinis* | 0 (0.0) | 0 (0.0) | 1 (0.8) | 1 (0.2) |
| *Streptococcus dysgalactiae* \| *Streptococcus mitis/oralis* \| *Streptococcus salivarius* | 0 (0.0) | 1 (1.2) | 0 (0.0) | 1 (0.2) |
| *Streptococcus gordonii* \| *Streptococcus salivarius* | 1 (0.3) | 0 (0.0) | 0 (0.0) | 1 (0.2) |
| *Streptococcus mitis/oralis* \| *Streptococcus parasanguinis* | 1 (0.3) | 0 (0.0) | 0 (0.0) | 1 (0.2) |
| *Streptococcus mitis/oralis* \| *Streptococcus parasanguinis* \| *Streptococcus salivarius* | 0 (0.0) | 0 (0.0) | 1 (0.8) | 1 (0.2) |
| *Streptococcus mitis/oralis* \| *Streptococcus viridans* | 1 (0.3) | 0 (0.0) | 0 (0.0) | 1 (0.2) |
| *Streptococcus parasanguinis* \| *Streptococcus sanguinis* | 1 (0.3) | 0 (0.0) | 0 (0.0) | 1 (0.2) |

The pre-restriction, restriction, and post-restriction periods were June 26, 2023–June 25, 2024, June 26, 2024–December 23, 2024, and December 24, 2024–June 25, 2025, respectively. Data shown are the number of bloodstream infectious episodes with the listed organism(s), with percentage in parentheses reflecting this as the numerator and size of the overall group as the denominator (shown in the headings). Single-organism episodes are listed first in order from most to least frequent, followed by multi-organism episodes in order from most to least frequent.

# **Supplementary Table 9.** Frequency of Individual Organisms Categorized as Rare Organisms in Bloodstream Infection Episodes Aggregated by Time Period.

| **Rare organisms, No. (%)** | **Pre-restriction**  **(N = 21)** | **Restriction**  **(N = 6)** | **Post-restriction**  **(N = 3)** | **Overall**  **(N = 30)** |
| --- | --- | --- | --- | --- |
| *Bacillus cereus* | 8 (38.1) | 1 (16.7) | 2 (66.7) | 11 (36.7) |
| *Arcanobacterium haemolyticum* | 5 (23.8) | 2 (33.3) | 0 (0.0) | 7 (23.3) |
| *Mycobacterium mucogenicum* | 2 (9.5) | 1 (16.7) | 0 (0.0) | 3 (10.0) |
| Gordonia species | 1 (4.8) | 1 (16.7) | 0 (0.0) | 2 (6.7) |
| *Mycobacterium fortuitum* group | 1 (4.8) | 1 (16.7) | 0 (0.0) | 2 (6.7) |
| Cruoricaptor species | 1 (4.8) | 0 (0.0) | 0 (0.0) | 1 (3.3) |
| *Gardnerella vaginalis* | 0 (0.0) | 0 (0.0) | 1 (33.3) | 1 (3.3) |
| *Mycobacterium avium-intracellulare* complex | 1 (4.8) | 0 (0.0) | 0 (0.0) | 1 (3.3) |
| Mycobacterium species | 1 (4.8) | 0 (0.0) | 0 (0.0) | 1 (3.3) |
| Paenibacillus species | 1 (4.8) | 0 (0.0) | 0 (0.0) | 1 (3.3) |

The pre-restriction, restriction, and post-restriction periods were June 26, 2023–June 25, 2024, June 26, 2024–December 23, 2024, and December 24, 2024–June 25, 2025, respectively. Data shown are the number of bloodstream infectious episodes with the listed organism(s), with percentage in parentheses reflecting this as the numerator and size of the overall group as the denominator (shown in the headings). Single-organism episodes are listed first in order from most to least frequent, followed by multi-organism episodes in order from most to least frequent.

# **Supplementary Table 10.** Frequency of Individual Organisms Categorized as Coagulase-Negative Staphylococci or Common Commensals in Bloodstream Infection Episodes Aggregated by Time Period.

| **Coagulase-negative staphylococci or common commensals, No. (%)** | **Pre-restriction**  **(N = 1 010)** | **Restriction**  **(N = 297)** | **Post-restriction**  **(N = 392)** | **Overall**  **(N = 1 699)** |
| --- | --- | --- | --- | --- |
| *Staphylococcus epidermidis* | 496 (49.1) | 167 (56.2) | 185 (47.2) | 848 (49.9) |
| *Staphylococcus hominis* | 136 (13.5) | 29 (9.8) | 60 (15.3) | 225 (13.2) |
| *Staphylococcus capitis* | 63 (6.2) | 14 (4.7) | 23 (5.9) | 100 (5.9) |
| Staphylococcus species, coagulase-negative | 43 (4.3) | 13 (4.4) | 14 (3.6) | 70 (4.1) |
| *Staphylococcus haemolyticus* | 30 (3.0) | 18 (6.1) | 16 (4.1) | 64 (3.8) |
| Corynebacterium species | 30 (3.0) | 7 (2.4) | 13 (3.3) | 50 (2.9) |
| Bacillus species | 15 (1.5) | 4 (1.3) | 6 (1.5) | 25 (1.5) |
| Micrococcus species | 17 (1.7) | 2 (0.7) | 3 (0.8) | 22 (1.3) |
| Diphtheroids | 13 (1.3) | 3 (1.0) | 3 (0.8) | 19 (1.1) |
| Gram positive bacilli, aerobic | 12 (1.2) | 2 (0.7) | 5 (1.3) | 19 (1.1) |
| Lactobacillus species | 11 (1.1) | 2 (0.7) | 4 (1.0) | 17 (1.0) |
| *Staphylococcus warneri* | 7 (0.7) | 2 (0.7) | 4 (1.0) | 13 (0.8) |
| *Cutibacterium acnes* | 3 (0.3) | 2 (0.7) | 5 (1.3) | 10 (0.6) |
| *Bacillus subtilis* | 6 (0.6) | 0 (0.0) | 1 (0.3) | 7 (0.4) |
| *Micrococcus luteus* | 5 (0.5) | 1 (0.3) | 0 (0.0) | 6 (0.4) |
| *Corynebacterium striatum* | 1 (0.1) | 3 (1.0) | 1 (0.3) | 5 (0.3) |
| *Rothia mucilaginosa* | 4 (0.4) | 0 (0.0) | 1 (0.3) | 5 (0.3) |
| *Staphylococcus cohnii* | 3 (0.3) | 1 (0.3) | 1 (0.3) | 5 (0.3) |
| *Corynebacterium jeikeium* | 2 (0.2) | 0 (0.0) | 2 (0.5) | 4 (0.2) |
| Kocuria species | 2 (0.2) | 1 (0.3) | 1 (0.3) | 4 (0.2) |
| *Staphylococcus simulans* | 2 (0.2) | 1 (0.3) | 1 (0.3) | 4 (0.2) |
| Brevibacillus species | 2 (0.2) | 0 (0.0) | 1 (0.3) | 3 (0.2) |
| Gulosibacter species | 0 (0.0) | 3 (1.0) | 0 (0.0) | 3 (0.2) |
| Rothia species | 1 (0.1) | 0 (0.0) | 2 (0.5) | 3 (0.2) |
| Dermabacter species | 1 (0.1) | 0 (0.0) | 1 (0.3) | 2 (0.1) |
| Gram positive bacilli | 2 (0.2) | 0 (0.0) | 0 (0.0) | 2 (0.1) |
| Gram positive cocci | 2 (0.2) | 0 (0.0) | 0 (0.0) | 2 (0.1) |
| *Staphylococcus saprophyticus* | 2 (0.2) | 0 (0.0) | 0 (0.0) | 2 (0.1) |
| *Brevibacterium casei* | 1 (0.1) | 0 (0.0) | 0 (0.0) | 1 (0.1) |
| Brevibacterium species | 1 (0.1) | 0 (0.0) | 0 (0.0) | 1 (0.1) |
| Gram variable bacilli | 0 (0.0) | 0 (0.0) | 1 (0.3) | 1 (0.1) |
| *Kocuria rhizophila* | 1 (0.1) | 0 (0.0) | 0 (0.0) | 1 (0.1) |
| *Lactobacillus jensenii* | 1 (0.1) | 0 (0.0) | 0 (0.0) | 1 (0.1) |
| *Staphylococcus auricularis* | 0 (0.0) | 1 (0.3) | 0 (0.0) | 1 (0.1) |
| *Staphylococcus schleiferi* | 1 (0.1) | 0 (0.0) | 0 (0.0) | 1 (0.1) |
| Tetragenococcus species | 1 (0.1) | 0 (0.0) | 0 (0.0) | 1 (0.1) |
| *Staphylococcus epidermidis* \| *Staphylococcus hominis* | 30 (3.0) | 7 (2.4) | 13 (3.3) | 50 (2.9) |
| *Staphylococcus epidermidis* \| *Staphylococcus haemolyticus* | 9 (0.9) | 0 (0.0) | 6 (1.5) | 15 (0.9) |
| *Staphylococcus capitis* \| *Staphylococcus epidermidis* | 6 (0.6) | 2 (0.7) | 4 (1.0) | 12 (0.7) |
| Corynebacterium species \| *Staphylococcus epidermidis* | 5 (0.5) | 1 (0.3) | 2 (0.5) | 8 (0.5) |
| *Staphylococcus capitis* \| *Staphylococcus hominis* | 3 (0.3) | 3 (1.0) | 1 (0.3) | 7 (0.4) |
| *Staphylococcus haemolyticus* \| *Staphylococcus hominis* | 3 (0.3) | 0 (0.0) | 1 (0.3) | 4 (0.2) |
| *Staphylococcus epidermidis* \| *Staphylococcus hominis* \| *Staphylococcus simulans* | 2 (0.2) | 0 (0.0) | 1 (0.3) | 3 (0.2) |
| *Staphylococcus epidermidis* \| *Staphylococcus warneri* | 3 (0.3) | 0 (0.0) | 0 (0.0) | 3 (0.2) |
| Corynebacterium species \| *Staphylococcus simulans* | 1 (0.1) | 0 (0.0) | 1 (0.3) | 2 (0.1) |
| *Corynebacterium striatum* \| *Staphylococcus epidermidis* | 2 (0.2) | 0 (0.0) | 0 (0.0) | 2 (0.1) |
| *Staphylococcus capitis* \| *Staphylococcus epidermidis* \| *Staphylococcus hominis* | 1 (0.1) | 1 (0.3) | 0 (0.0) | 2 (0.1) |
| *Staphylococcus capitis* \| *Staphylococcus haemolyticus* | 2 (0.2) | 0 (0.0) | 0 (0.0) | 2 (0.1) |
| *Staphylococcus cohnii* \| *Staphylococcus epidermidis* | 1 (0.1) | 0 (0.0) | 1 (0.3) | 2 (0.1) |
| *Staphylococcus epidermidis* \| *Staphylococcus haemolyticus* \| *Staphylococcus hominis* | 1 (0.1) | 0 (0.0) | 1 (0.3) | 2 (0.1) |
| *Staphylococcus hominis* \| *Staphylococcus warneri* | 2 (0.2) | 0 (0.0) | 0 (0.0) | 2 (0.1) |
| Bacillus species \| *Staphylococcus hominis* | 1 (0.1) | 0 (0.0) | 0 (0.0) | 1 (0.1) |
| Corynebacterium species \| Dermabacter species | 0 (0.0) | 1 (0.3) | 0 (0.0) | 1 (0.1) |
| Corynebacterium species \| Diphtheroids | 1 (0.1) | 0 (0.0) | 0 (0.0) | 1 (0.1) |
| Corynebacterium species \| *Staphylococcus capitis* \| *Staphylococcus epidermidis* | 0 (0.0) | 0 (0.0) | 1 (0.3) | 1 (0.1) |
| Corynebacterium species \| *Staphylococcus capitis* \| *Staphylococcus hominis* | 0 (0.0) | 0 (0.0) | 1 (0.3) | 1 (0.1) |
| Corynebacterium species \| *Staphylococcus epidermidis* \| *Staphylococcus hominis* | 0 (0.0) | 0 (0.0) | 1 (0.3) | 1 (0.1) |
| Corynebacterium species \| *Staphylococcus haemolyticus* | 1 (0.1) | 0 (0.0) | 0 (0.0) | 1 (0.1) |
| Corynebacterium species \| *Staphylococcus hominis* | 1 (0.1) | 0 (0.0) | 0 (0.0) | 1 (0.1) |
| *Corynebacterium striatum* \| *Staphylococcus capitis* | 1 (0.1) | 0 (0.0) | 0 (0.0) | 1 (0.1) |
| *Cutibacterium acnes* \| *Staphylococcus epidermidis* | 0 (0.0) | 1 (0.3) | 0 (0.0) | 1 (0.1) |
| *Cutibacterium acnes* \| Staphylococcus species, coagulase-negative | 0 (0.0) | 1 (0.3) | 0 (0.0) | 1 (0.1) |
| *Cutibacterium avidum* \| *Staphylococcus capitis* | 1 (0.1) | 0 (0.0) | 0 (0.0) | 1 (0.1) |
| *Cutibacterium avidum* \| *Staphylococcus epidermidis* | 1 (0.1) | 0 (0.0) | 0 (0.0) | 1 (0.1) |
| Dermabacter species \| *Staphylococcus haemolyticus* | 1 (0.1) | 0 (0.0) | 0 (0.0) | 1 (0.1) |
| Diphtheroids \| *Staphylococcus epidermidis* | 0 (0.0) | 0 (0.0) | 1 (0.3) | 1 (0.1) |
| Diphtheroids \| *Staphylococcus simulans* | 1 (0.1) | 0 (0.0) | 0 (0.0) | 1 (0.1) |
| Kocuria species \| *Staphylococcus epidermidis* \| *Staphylococcus hominis* | 0 (0.0) | 0 (0.0) | 1 (0.3) | 1 (0.1) |
| *Micrococcus luteus* \| *Staphylococcus hominis* | 0 (0.0) | 0 (0.0) | 1 (0.3) | 1 (0.1) |
| *Micrococcus luteus* \| *Staphylococcus simulans* | 1 (0.1) | 0 (0.0) | 0 (0.0) | 1 (0.1) |
| Micrococcus species \| *Staphylococcus capitis* | 1 (0.1) | 0 (0.0) | 0 (0.0) | 1 (0.1) |
| Micrococcus species \| *Staphylococcus epidermidis* | 0 (0.0) | 1 (0.3) | 0 (0.0) | 1 (0.1) |
| Pseudoglutamicibacter species \| Staphylococcus species, coagulase-negative | 1 (0.1) | 0 (0.0) | 0 (0.0) | 1 (0.1) |
| *Rothia kristinae* \| *Staphylococcus capitis* | 1 (0.1) | 0 (0.0) | 0 (0.0) | 1 (0.1) |
| *Rothia mucilaginosa* \| *Staphylococcus epidermidis* \| *Staphylococcus simulans* | 1 (0.1) | 0 (0.0) | 0 (0.0) | 1 (0.1) |
| Rothia species \| Staphylococcus species, coagulase-negative | 1 (0.1) | 0 (0.0) | 0 (0.0) | 1 (0.1) |
| *Staphylococcus auricularis* \| *Staphylococcus epidermidis* | 0 (0.0) | 1 (0.3) | 0 (0.0) | 1 (0.1) |
| *Staphylococcus capitis* \| *Staphylococcus cohnii* | 1 (0.1) | 0 (0.0) | 0 (0.0) | 1 (0.1) |
| *Staphylococcus capitis* \| *Staphylococcus cohnii* \| *Staphylococcus epidermidis* | 1 (0.1) | 0 (0.0) | 0 (0.0) | 1 (0.1) |
| *Staphylococcus capitis* \| *Staphylococcus epidermidis* \| *Staphylococcus warneri* | 1 (0.1) | 0 (0.0) | 0 (0.0) | 1 (0.1) |
| *Staphylococcus capitis* \| *Staphylococcus saprophyticus* | 0 (0.0) | 1 (0.3) | 0 (0.0) | 1 (0.1) |
| *Staphylococcus caprae* \| Staphylococcus species, coagulase-negative | 1 (0.1) | 0 (0.0) | 0 (0.0) | 1 (0.1) |
| *Staphylococcus cohnii* \| *Staphylococcus epidermidis* \| *Staphylococcus hominis* | 1 (0.1) | 0 (0.0) | 0 (0.0) | 1 (0.1) |
| *Staphylococcus epidermidis* \| *Staphylococcus hominis* \| *Staphylococcus warneri* | 0 (0.0) | 0 (0.0) | 1 (0.3) | 1 (0.1) |
| *Staphylococcus epidermidis* \| *Staphylococcus simulans* \| Staphylococcus species, coagulase-negative | 0 (0.0) | 1 (0.3) | 0 (0.0) | 1 (0.1) |
| *Staphylococcus haemolyticus* \| *Staphylococcus simulans* | 1 (0.1) | 0 (0.0) | 0 (0.0) | 1 (0.1) |
| *Staphylococcus simulans* \| *Staphylococcus warneri* | 1 (0.1) | 0 (0.0) | 0 (0.0) | 1 (0.1) |

The pre-restriction, restriction, and post-restriction periods were June 26, 2023–June 25, 2024, June 26, 2024–December 23, 2024, and December 24, 2024–June 25, 2025, respectively. Data shown are the number of bloodstream infectious episodes with the listed organism(s), with percentage in parentheses reflecting this as the numerator and size of the overall group as the denominator (shown in the headings). Single-organism episodes are listed first in order from most to least frequent, followed by multi-organism episodes in order from most to least frequent.

# **Supplementary Table 11.** Frequency of Individual Organisms Categorized as Polymicrobial Bloodstream Infection Episodes Aggregated by Time Period.

| **Polymicrobial episodes, No. (%)** | **Pre-restriction**  **(N = 152)** | **Restriction**  **(N = 34)** | **Post-restriction**  **(N = 83)** | **Overall**  **(N = 269)** |
| --- | --- | --- | --- | --- |
| *Enterococcus faecalis* \| *Escherichia coli* | 1 (0.7) | 1 (2.9) | 3 (3.6) | 5 (1.9) |
| *Enterococcus faecalis* \| *Staphylococcus aureus*, methicillin-susceptible | 2 (1.3) | 2 (5.9) | 1 (1.2) | 5 (1.9) |
| *Candida glabrata* \| *Klebsiella pneumoniae* | 2 (1.3) | 0 (0.0) | 2 (2.4) | 4 (1.5) |
| *Enterococcus faecalis* \| *Staphylococcus aureus*, methicillin-resistant | 0 (0.0) | 1 (2.9) | 3 (3.6) | 4 (1.5) |
| *Staphylococcus aureus*, methicillin-resistant \| *Streptococcus pyogenes* | 3 (2.0) | 1 (2.9) | 0 (0.0) | 4 (1.5) |
| *Bacteroides fragilis* \| *Escherichia coli* | 3 (2.0) | 0 (0.0) | 0 (0.0) | 3 (1.1) |
| *Enterococcus faecalis* \| *Klebsiella pneumoniae* | 1 (0.7) | 0 (0.0) | 2 (2.4) | 3 (1.1) |
| *Enterococcus faecium* \| *Escherichia coli* | 1 (0.7) | 0 (0.0) | 2 (2.4) | 3 (1.1) |
| *Enterococcus faecium* \| *Klebsiella pneumoniae* | 1 (0.7) | 2 (5.9) | 0 (0.0) | 3 (1.1) |
| *Pseudomonas aeruginosa* \| *Staphylococcus aureus*, methicillin-susceptible | 2 (1.3) | 0 (0.0) | 1 (1.2) | 3 (1.1) |
| *Staphylococcus aureus*, methicillin-susceptible \| *Streptococcus agalactiae* | 0 (0.0) | 1 (2.9) | 2 (2.4) | 3 (1.1) |
| *Bacteroides fragilis* \| *Streptococcus constellatus* | 1 (0.7) | 0 (0.0) | 1 (1.2) | 2 (0.7) |
| *Bacteroides thetaiotaomicron* \| *Enterococcus faecalis* | 1 (0.7) | 1 (2.9) | 0 (0.0) | 2 (0.7) |
| *Candida albicans* \| *Staphylococcus aureus*, methicillin-resistant | 1 (0.7) | 0 (0.0) | 1 (1.2) | 2 (0.7) |
| *Candida albicans* \| *Staphylococcus aureus*, methicillin-susceptible | 1 (0.7) | 0 (0.0) | 1 (1.2) | 2 (0.7) |
| *Candida parapsilosis* \| *Enterococcus faecalis* | 1 (0.7) | 0 (0.0) | 1 (1.2) | 2 (0.7) |
| *Candida parapsilosis* \| *Klebsiella pneumoniae* | 2 (1.3) | 0 (0.0) | 0 (0.0) | 2 (0.7) |
| *Enterobacter cloacae* complex \| *Enterococcus faecium* | 1 (0.7) | 1 (2.9) | 0 (0.0) | 2 (0.7) |
| *Enterobacter cloacae* complex \| *Staphylococcus aureus*, methicillin-susceptible | 1 (0.7) | 0 (0.0) | 1 (1.2) | 2 (0.7) |
| *Enterococcus faecalis* \| *Morganella morganii* | 0 (0.0) | 1 (2.9) | 1 (1.2) | 2 (0.7) |
| *Enterococcus faecalis* \| *Parabacteroides distasonis* | 2 (1.3) | 0 (0.0) | 0 (0.0) | 2 (0.7) |
| *Enterococcus faecalis* \| *Pseudomonas aeruginosa* | 2 (1.3) | 0 (0.0) | 0 (0.0) | 2 (0.7) |
| *Enterococcus faecalis* \| *Streptococcus mitis/oralis* | 2 (1.3) | 0 (0.0) | 0 (0.0) | 2 (0.7) |
| *Enterococcus faecium* \| *Streptococcus anginosus* | 1 (0.7) | 0 (0.0) | 1 (1.2) | 2 (0.7) |
| *Enterococcus faecium* \| *Streptococcus mitis/oralis* | 1 (0.7) | 0 (0.0) | 1 (1.2) | 2 (0.7) |
| *Escherichia coli* \| *Streptococcus mitis/oralis* | 1 (0.7) | 1 (2.9) | 0 (0.0) | 2 (0.7) |
| *Escherichia coli* \| *Streptococcus pyogenes* | 2 (1.3) | 0 (0.0) | 0 (0.0) | 2 (0.7) |
| *Escherichia coli* \| *Streptococcus sanguinis* | 0 (0.0) | 1 (2.9) | 1 (1.2) | 2 (0.7) |
| *Klebsiella pneumoniae* \| *Staphylococcus aureus*, methicillin-resistant | 2 (1.3) | 0 (0.0) | 0 (0.0) | 2 (0.7) |
| *Klebsiella pneumoniae* \| *Staphylococcus aureus*, methicillin-susceptible | 2 (1.3) | 0 (0.0) | 0 (0.0) | 2 (0.7) |
| *Klebsiella pneumoniae* \| *Streptococcus pyogenes* | 2 (1.3) | 0 (0.0) | 0 (0.0) | 2 (0.7) |
| *Morganella morganii* \| *Staphylococcus aureus*, methicillin-resistant | 1 (0.7) | 0 (0.0) | 1 (1.2) | 2 (0.7) |
| *Proteus mirabilis* \| *Staphylococcus aureus*, methicillin-resistant | 2 (1.3) | 0 (0.0) | 0 (0.0) | 2 (0.7) |
| *Staphylococcus aureus*, methicillin-resistant \| *Streptococcus dysgalactiae* | 2 (1.3) | 0 (0.0) | 0 (0.0) | 2 (0.7) |
| *Staphylococcus aureus*, methicillin-susceptible \| *Streptococcus pyogenes* | 2 (1.3) | 0 (0.0) | 0 (0.0) | 2 (0.7) |
| *Abiotrophia defectiva* \| *Pseudomonas aeruginosa* | 0 (0.0) | 0 (0.0) | 1 (1.2) | 1 (0.4) |
| Achromobacter species \| *Candida tropicalis* \| *Enterococcus faecium* | 1 (0.7) | 0 (0.0) | 0 (0.0) | 1 (0.4) |
| Achromobacter species \| *Clostridium ramosum* | 0 (0.0) | 0 (0.0) | 1 (1.2) | 1 (0.4) |
| *Acinetobacter baumannii* complex \| *Aerococcus viridans* \| *Psychrobacter phenylpyruvicus* | 0 (0.0) | 0 (0.0) | 1 (1.2) | 1 (0.4) |
| *Acinetobacter baumannii* complex \| *Clostridium septicum* \| *Enterococcus faecium* \| *Pseudomonas aeruginosa* | 1 (0.7) | 0 (0.0) | 0 (0.0) | 1 (0.4) |
| *Acinetobacter baumannii* complex \| *Enterococcus faecalis* | 0 (0.0) | 0 (0.0) | 1 (1.2) | 1 (0.4) |
| *Acinetobacter baumannii* complex \| *Enterococcus faecium* \| *Pseudomonas aeruginosa* | 1 (0.7) | 0 (0.0) | 0 (0.0) | 1 (0.4) |
| *Acinetobacter baumannii* complex \| *Pseudomonas aeruginosa* \| *Staphylococcus aureus*, methicillin-susceptible | 1 (0.7) | 0 (0.0) | 0 (0.0) | 1 (0.4) |
| *Acinetobacter baumannii* complex \| *Streptococcus dysgalactiae* | 1 (0.7) | 0 (0.0) | 0 (0.0) | 1 (0.4) |
| Acinetobacter species \| *Candida albicans* \| *Staphylococcus aureus*, methicillin-susceptible \| *Streptococcus anginosus* \| *Streptococcus mitis/oralis* | 0 (0.0) | 1 (2.9) | 0 (0.0) | 1 (0.4) |
| Actinomyces species \| *Bacteroides fragilis* \| *Stenotrophomonas maltophilia* | 0 (0.0) | 0 (0.0) | 1 (1.2) | 1 (0.4) |
| *Actinotignum schaalii* \| Bifidobacterium species \| *Streptococcus anginosus* | 0 (0.0) | 0 (0.0) | 1 (1.2) | 1 (0.4) |
| *Actinotignum schaalii* \| *Enterobacter cloacae* complex | 0 (0.0) | 0 (0.0) | 1 (1.2) | 1 (0.4) |
| *Actinotignum schaalii* \| *Escherichia coli* | 1 (0.7) | 0 (0.0) | 0 (0.0) | 1 (0.4) |
| *Aerococcus viridans* \| *Klebsiella pneumoniae* \| *Streptococcus viridans* | 0 (0.0) | 1 (2.9) | 0 (0.0) | 1 (0.4) |
| Alcaligenes species \| *Bacteroides fragilis* \| *Citrobacter freundii* complex \| *Proteus mirabilis* \| *Pseudomonas aeruginosa* | 1 (0.7) | 0 (0.0) | 0 (0.0) | 1 (0.4) |
| Alcaligenes species \| *Staphylococcus aureus*, methicillin-resistant | 0 (0.0) | 0 (0.0) | 1 (1.2) | 1 (0.4) |
| *Arcanobacterium haemolyticum* \| *Staphylococcus epidermidis* | 1 (0.7) | 0 (0.0) | 0 (0.0) | 1 (0.4) |
| *Bacillus cereus* \| *Staphylococcus haemolyticus* | 1 (0.7) | 0 (0.0) | 0 (0.0) | 1 (0.4) |
| *Bacillus cereus* \| *Staphylococcus hominis* | 0 (0.0) | 0 (0.0) | 1 (1.2) | 1 (0.4) |
| Bacillus species \| Paenibacillus species | 1 (0.7) | 0 (0.0) | 0 (0.0) | 1 (0.4) |
| *Bacteroides fragilis* \| *Candida albicans* \| *Candida glabrata* \| *Prevotella intermedia* | 1 (0.7) | 0 (0.0) | 0 (0.0) | 1 (0.4) |
| *Bacteroides fragilis* \| *Citrobacter farmeri* \| *Klebsiella pneumoniae* | 1 (0.7) | 0 (0.0) | 0 (0.0) | 1 (0.4) |
| *Bacteroides fragilis* \| *Clostridium innocuum* \| *Pseudomonas aeruginosa* | 1 (0.7) | 0 (0.0) | 0 (0.0) | 1 (0.4) |
| *Bacteroides fragilis* \| Eggerthella species \| *Pseudomonas aeruginosa* | 1 (0.7) | 0 (0.0) | 0 (0.0) | 1 (0.4) |
| *Bacteroides fragilis* \| *Eikenella corrodens* | 1 (0.7) | 0 (0.0) | 0 (0.0) | 1 (0.4) |
| *Bacteroides fragilis* \| *Enterobacter cloacae* complex \| *Enterococcus faecium* | 0 (0.0) | 1 (2.9) | 0 (0.0) | 1 (0.4) |
| *Bacteroides fragilis* \| *Escherichia coli* \| *Gemella morbillorum* | 0 (0.0) | 0 (0.0) | 1 (1.2) | 1 (0.4) |
| *Bacteroides fragilis* \| *Klebsiella pneumoniae* | 0 (0.0) | 0 (0.0) | 1 (1.2) | 1 (0.4) |
| *Bacteroides fragilis* \| *Parvimonas micra* \| Streptococcus species, beta-hemolytic, group C | 1 (0.7) | 0 (0.0) | 0 (0.0) | 1 (0.4) |
| *Bacteroides fragilis* \| *Prevotella bivia* \| *Staphylococcus aureus*, methicillin-susceptible | 1 (0.7) | 0 (0.0) | 0 (0.0) | 1 (0.4) |
| *Bacteroides fragilis* \| *Pseudomonas aeruginosa* | 0 (0.0) | 0 (0.0) | 1 (1.2) | 1 (0.4) |
| *Bacteroides fragilis* \| *Staphylococcus aureus*, methicillin-resistant \| *Streptococcus dysgalactiae* \| *Streptococcus mitis/oralis* | 1 (0.7) | 0 (0.0) | 0 (0.0) | 1 (0.4) |
| *Bacteroides fragilis* \| *Streptococcus anginosus* \| *Streptococcus constellatus* | 0 (0.0) | 0 (0.0) | 1 (1.2) | 1 (0.4) |
| *Bacteroides fragilis* \| *Streptococcus mitis/oralis* | 1 (0.7) | 0 (0.0) | 0 (0.0) | 1 (0.4) |
| Bacteroides species \| *Klebsiella aerogenes* | 1 (0.7) | 0 (0.0) | 0 (0.0) | 1 (0.4) |
| *Bacteroides thetaiotaomicron* \| *Bacteroides uniformis* \| *Enterococcus faecalis* | 0 (0.0) | 0 (0.0) | 1 (1.2) | 1 (0.4) |
| *Bacteroides thetaiotaomicron* \| *Candida albicans* | 0 (0.0) | 0 (0.0) | 1 (1.2) | 1 (0.4) |
| *Bacteroides thetaiotaomicron* \| *Candida albicans* \| *Enterococcus faecalis* | 0 (0.0) | 0 (0.0) | 1 (1.2) | 1 (0.4) |
| *Bacteroides thetaiotaomicron* \| *Candida tropicalis* \| *Enterococcus faecium* \| *Staphylococcus aureus*, methicillin-susceptible | 1 (0.7) | 0 (0.0) | 0 (0.0) | 1 (0.4) |
| *Bacteroides thetaiotaomicron* \| *Escherichia coli* | 1 (0.7) | 0 (0.0) | 0 (0.0) | 1 (0.4) |
| *Bacteroides thetaiotaomicron* \| *Escherichia coli* \| *Staphylococcus aureus*, methicillin-susceptible | 1 (0.7) | 0 (0.0) | 0 (0.0) | 1 (0.4) |
| *Bacteroides vulgatus* \| Peptostreptococcus species \| *Streptococcus infantarius* | 0 (0.0) | 1 (2.9) | 0 (0.0) | 1 (0.4) |
| Bifidobacterium species \| *Enterococcus faecium* \| *Pseudomonas aeruginosa* | 1 (0.7) | 0 (0.0) | 0 (0.0) | 1 (0.4) |
| Bifidobacterium species \| *Streptococcus constellatus* | 1 (0.7) | 0 (0.0) | 0 (0.0) | 1 (0.4) |
| *Bordetella trematum* \| *Morganella morganii* \| *Streptococcus dysgalactiae* | 0 (0.0) | 0 (0.0) | 1 (1.2) | 1 (0.4) |
| *Burkholderia cepacia* complex \| *Candida glabrata* \| *Klebsiella pneumoniae* \| *Staphylococcus aureus*, methicillin-susceptible \| *Streptococcus viridans* | 1 (0.7) | 0 (0.0) | 0 (0.0) | 1 (0.4) |
| *Candida albicans* \| *Candida glabrata* \| *Candida kefyr* \| *Enterococcus faecium* | 1 (0.7) | 0 (0.0) | 0 (0.0) | 1 (0.4) |
| *Candida albicans* \| *Candida glabrata* \| *Enterococcus faecium* | 1 (0.7) | 0 (0.0) | 0 (0.0) | 1 (0.4) |
| *Candida albicans* \| *Enterobacter cloacae* complex | 0 (0.0) | 1 (2.9) | 0 (0.0) | 1 (0.4) |
| *Candida albicans* \| *Enterococcus faecalis* | 1 (0.7) | 0 (0.0) | 0 (0.0) | 1 (0.4) |
| *Candida albicans* \| *Enterococcus faecalis* \| *Escherichia coli* \| *Klebsiella pneumoniae* | 0 (0.0) | 0 (0.0) | 1 (1.2) | 1 (0.4) |
| *Candida albicans* \| *Enterococcus faecalis* \| *Klebsiella oxytoca* \| *Klebsiella pneumoniae* | 1 (0.7) | 0 (0.0) | 0 (0.0) | 1 (0.4) |
| *Candida albicans* \| *Enterococcus faecium* | 1 (0.7) | 0 (0.0) | 0 (0.0) | 1 (0.4) |
| *Candida albicans* \| *Escherichia coli* | 1 (0.7) | 0 (0.0) | 0 (0.0) | 1 (0.4) |
| *Candida albicans* \| Gemella species | 1 (0.7) | 0 (0.0) | 0 (0.0) | 1 (0.4) |
| *Candida albicans* \| *Klebsiella pneumoniae* | 1 (0.7) | 0 (0.0) | 0 (0.0) | 1 (0.4) |
| *Candida auris* \| *Escherichia coli* | 0 (0.0) | 1 (2.9) | 0 (0.0) | 1 (0.4) |
| *Candida dubliniensis* \| *Candida glabrata* \| *Candida tropicalis* \| *Enterobacter cloacae* complex \| *Enterococcus faecalis* \| *Klebsiella oxytoca* \| *Pseudomonas aeruginosa* \| *Staphylococcus aureus*, methicillin-resistant \| *Streptococcus mitis/oralis* | 0 (0.0) | 1 (2.9) | 0 (0.0) | 1 (0.4) |
| *Candida dubliniensis* \| *Escherichia coli* | 1 (0.7) | 0 (0.0) | 0 (0.0) | 1 (0.4) |
| *Candida glabrata* \| *Candida lusitaniae* \| *Citrobacter freundii* complex \| *Enterobacter cloacae* complex \| *Escherichia coli* \| *Klebsiella oxytoca* \| Lactococcus species \| *Pseudomonas aeruginosa* \| *Pseudomonas fluorescens* \| *Raoultella ornithinolytica* \| *Stenotrophomonas maltophilia* | 1 (0.7) | 0 (0.0) | 0 (0.0) | 1 (0.4) |
| *Candida glabrata* \| *Candida parapsilosis* \| *Enterobacter cloacae* complex \| *Escherichia coli* | 0 (0.0) | 0 (0.0) | 1 (1.2) | 1 (0.4) |
| *Candida glabrata* \| *Enterobacter cloacae* complex \| *Enterococcus faecium* \| *Escherichia coli* \| *Klebsiella pneumoniae* \| *Veillonella dispar* | 1 (0.7) | 0 (0.0) | 0 (0.0) | 1 (0.4) |
| *Candida glabrata* \| *Enterobacter cloacae* complex \| *Staphylococcus aureus*, methicillin-resistant | 0 (0.0) | 0 (0.0) | 1 (1.2) | 1 (0.4) |
| *Candida glabrata* \| *Enterococcus faecalis* | 0 (0.0) | 0 (0.0) | 1 (1.2) | 1 (0.4) |
| *Candida glabrata* \| *Enterococcus faecalis* \| *Enterococcus faecium* \| *Escherichia coli* \| *Klebsiella pneumoniae* \| *Pseudomonas aeruginosa* | 0 (0.0) | 0 (0.0) | 1 (1.2) | 1 (0.4) |
| *Candida glabrata* \| *Enterococcus faecalis* \| *Klebsiella pneumoniae* | 0 (0.0) | 0 (0.0) | 1 (1.2) | 1 (0.4) |
| *Candida glabrata* \| *Enterococcus faecium* \| *Pseudomonas aeruginosa* | 0 (0.0) | 1 (2.9) | 0 (0.0) | 1 (0.4) |
| *Candida glabrata* \| *Klebsiella pneumoniae* \| *Staphylococcus aureus*, methicillin-resistant | 1 (0.7) | 0 (0.0) | 0 (0.0) | 1 (0.4) |
| *Candida glabrata* \| Pantoea species | 1 (0.7) | 0 (0.0) | 0 (0.0) | 1 (0.4) |
| *Candida glabrata* \| *Pseudomonas aeruginosa* | 0 (0.0) | 0 (0.0) | 1 (1.2) | 1 (0.4) |
| *Candida guilliermondii* \| *Candida krusei* \| *Klebsiella pneumoniae* | 1 (0.7) | 0 (0.0) | 0 (0.0) | 1 (0.4) |
| *Candida guilliermondii* \| *Enterococcus casseliflavus* \| *Klebsiella pneumoniae* \| *Pantoea agglomerans* | 1 (0.7) | 0 (0.0) | 0 (0.0) | 1 (0.4) |
| *Candida guilliermondii* \| *Enterococcus faecium* \| *Klebsiella pneumoniae* | 0 (0.0) | 0 (0.0) | 1 (1.2) | 1 (0.4) |
| *Candida lusitaniae* \| *Enterococcus faecalis* \| *Staphylococcus aureus*, methicillin-resistant | 1 (0.7) | 0 (0.0) | 0 (0.0) | 1 (0.4) |
| *Candida parapsilosis* \| *Enterococcus avium* | 1 (0.7) | 0 (0.0) | 0 (0.0) | 1 (0.4) |
| *Candida parapsilosis* \| *Enterococcus faecalis* \| *Escherichia coli* | 1 (0.7) | 0 (0.0) | 0 (0.0) | 1 (0.4) |
| *Candida parapsilosis* \| *Escherichia coli* | 1 (0.7) | 0 (0.0) | 0 (0.0) | 1 (0.4) |
| *Candida parapsilosis* \| *Proteus mirabilis* \| Roseomonas species | 1 (0.7) | 0 (0.0) | 0 (0.0) | 1 (0.4) |
| *Candida parapsilosis* \| *Serratia marcescens* | 0 (0.0) | 0 (0.0) | 1 (1.2) | 1 (0.4) |
| *Candida parapsilosis* \| *Streptococcus dysgalactiae* | 0 (0.0) | 0 (0.0) | 1 (1.2) | 1 (0.4) |
| *Candida pelliculosa* \| Raoultella species | 1 (0.7) | 0 (0.0) | 0 (0.0) | 1 (0.4) |
| *Candida tropicalis* \| *Enterococcus faecalis* | 0 (0.0) | 1 (2.9) | 0 (0.0) | 1 (0.4) |
| *Candida tropicalis* \| *Enterococcus faecium* | 1 (0.7) | 0 (0.0) | 0 (0.0) | 1 (0.4) |
| *Citrobacter amalonaticus* \| *Enterococcus faecalis* \| *Escherichia coli* | 0 (0.0) | 0 (0.0) | 1 (1.2) | 1 (0.4) |
| *Citrobacter farmeri* \| *Enterococcus faecalis* | 1 (0.7) | 0 (0.0) | 0 (0.0) | 1 (0.4) |
| *Citrobacter freundii* complex \| *Enterobacter cloacae* complex \| *Enterococcus faecalis* \| *Klebsiella oxytoca* | 0 (0.0) | 0 (0.0) | 1 (1.2) | 1 (0.4) |
| *Citrobacter freundii* complex \| *Enterobacter cloacae* complex \| *Streptococcus anginosus* \| *Streptococcus constellatus* | 0 (0.0) | 0 (0.0) | 1 (1.2) | 1 (0.4) |
| *Citrobacter freundii* complex \| *Enterococcus faecalis* \| *Enterococcus faecium* | 0 (0.0) | 0 (0.0) | 1 (1.2) | 1 (0.4) |
| *Citrobacter freundii* complex \| *Staphylococcus aureus*, methicillin-susceptible | 0 (0.0) | 0 (0.0) | 1 (1.2) | 1 (0.4) |
| *Clostridium cadaveris* \| *Klebsiella pneumoniae* | 1 (0.7) | 0 (0.0) | 0 (0.0) | 1 (0.4) |
| *Clostridium perfringens* \| *Escherichia coli* | 0 (0.0) | 0 (0.0) | 1 (1.2) | 1 (0.4) |
| *Clostridium perfringens* \| *Escherichia coli* \| *Klebsiella pneumoniae* | 0 (0.0) | 0 (0.0) | 1 (1.2) | 1 (0.4) |
| *Clostridium ramosum* \| *Escherichia coli* | 0 (0.0) | 0 (0.0) | 1 (1.2) | 1 (0.4) |
| *Clostridium ramosum* \| *Streptococcus agalactiae* | 0 (0.0) | 1 (2.9) | 0 (0.0) | 1 (0.4) |
| *Clostridium septicum* \| *Enterococcus faecalis* | 1 (0.7) | 0 (0.0) | 0 (0.0) | 1 (0.4) |
| Clostridium species \| *Lacrimispora celerecrescens* \| *Stenotrophomonas maltophilia* | 0 (0.0) | 0 (0.0) | 1 (1.2) | 1 (0.4) |
| Clostridium species \| Moraxella species | 1 (0.7) | 0 (0.0) | 0 (0.0) | 1 (0.4) |
| *Clostridium tertium* \| *Enterococcus faecium* \| *Pseudomonas aeruginosa* | 1 (0.7) | 0 (0.0) | 0 (0.0) | 1 (0.4) |
| *Cryptococcus neoformans* \| *Enterobacter cloacae* complex | 0 (0.0) | 0 (0.0) | 1 (1.2) | 1 (0.4) |
| *Eggerthella lenta* \| *Escherichia coli* | 1 (0.7) | 0 (0.0) | 0 (0.0) | 1 (0.4) |
| *Enterobacter cloacae* complex \| *Enterococcus faecalis* | 1 (0.7) | 0 (0.0) | 0 (0.0) | 1 (0.4) |
| *Enterobacter cloacae* complex \| *Enterococcus faecalis* \| *Escherichia coli* \| *Pseudomonas aeruginosa* | 1 (0.7) | 0 (0.0) | 0 (0.0) | 1 (0.4) |
| *Enterobacter cloacae* complex \| *Enterococcus faecalis* \| *Serratia marcescens* | 1 (0.7) | 0 (0.0) | 0 (0.0) | 1 (0.4) |
| *Enterobacter cloacae* complex \| *Enterococcus faecium* \| *Klebsiella pneumoniae* \| *Streptococcus intermedius* | 1 (0.7) | 0 (0.0) | 0 (0.0) | 1 (0.4) |
| *Enterobacter cloacae* complex \| *Enterococcus faecium* \| *Providencia stuartii* | 1 (0.7) | 0 (0.0) | 0 (0.0) | 1 (0.4) |
| *Enterobacter cloacae* complex \| *Enterococcus gallinarum* | 1 (0.7) | 0 (0.0) | 0 (0.0) | 1 (0.4) |
| *Enterobacter cloacae* complex \| *Escherichia coli* \| *Klebsiella pneumoniae* \| *Streptococcus mitis/oralis* | 1 (0.7) | 0 (0.0) | 0 (0.0) | 1 (0.4) |
| *Enterobacter cloacae* complex \| *Staphylococcus lugdunensis* | 1 (0.7) | 0 (0.0) | 0 (0.0) | 1 (0.4) |
| *Enterococcus avium* \| *Escherichia coli* \| *Klebsiella pneumoniae* | 1 (0.7) | 0 (0.0) | 0 (0.0) | 1 (0.4) |
| *Enterococcus avium* \| *Escherichia coli* \| Kluyvera species | 1 (0.7) | 0 (0.0) | 0 (0.0) | 1 (0.4) |
| *Enterococcus avium* \| *Escherichia coli* \| *Proteus mirabilis* \| *Streptococcus anginosus* | 1 (0.7) | 0 (0.0) | 0 (0.0) | 1 (0.4) |
| *Enterococcus avium* \| *Proteus mirabilis* \| *Staphylococcus aureus*, methicillin-susceptible | 1 (0.7) | 0 (0.0) | 0 (0.0) | 1 (0.4) |
| *Enterococcus casseliflavus* \| *Klebsiella pneumoniae* \| *Pseudomonas aeruginosa* | 1 (0.7) | 0 (0.0) | 0 (0.0) | 1 (0.4) |
| *Enterococcus durans* \| *Staphylococcus aureus*, methicillin-susceptible | 1 (0.7) | 0 (0.0) | 0 (0.0) | 1 (0.4) |
| *Enterococcus faecalis* \| *Enterococcus faecium* \| *Streptococcus mitis/oralis* | 1 (0.7) | 0 (0.0) | 0 (0.0) | 1 (0.4) |
| *Enterococcus faecalis* \| *Escherichia coli* \| *Klebsiella oxytoca* \| *Klebsiella pneumoniae* | 1 (0.7) | 0 (0.0) | 0 (0.0) | 1 (0.4) |
| *Enterococcus faecalis* \| *Escherichia coli* \| *Klebsiella oxytoca* \| *Klebsiella pneumoniae* \| *Proteus mirabilis* \| *Pseudomonas aeruginosa* | 0 (0.0) | 0 (0.0) | 1 (1.2) | 1 (0.4) |
| *Enterococcus faecalis* \| *Escherichia coli* \| *Klebsiella pneumoniae* \| *Proteus mirabilis* | 0 (0.0) | 0 (0.0) | 1 (1.2) | 1 (0.4) |
| *Enterococcus faecalis* \| *Escherichia coli* \| *Klebsiella pneumoniae* \| *Pseudomonas aeruginosa* | 1 (0.7) | 0 (0.0) | 0 (0.0) | 1 (0.4) |
| *Enterococcus faecalis* \| *Escherichia coli* \| *Pseudomonas aeruginosa* | 1 (0.7) | 0 (0.0) | 0 (0.0) | 1 (0.4) |
| *Enterococcus faecalis* \| *Klebsiella pneumoniae* \| *Providencia stuartii* | 1 (0.7) | 0 (0.0) | 0 (0.0) | 1 (0.4) |
| *Enterococcus faecalis* \| *Klebsiella pneumoniae* \| *Staphylococcus aureus*, methicillin-susceptible | 0 (0.0) | 0 (0.0) | 1 (1.2) | 1 (0.4) |
| *Enterococcus faecalis* \| *Proteus mirabilis* | 1 (0.7) | 0 (0.0) | 0 (0.0) | 1 (0.4) |
| *Enterococcus faecalis* \| *Proteus mirabilis* \| *Staphylococcus aureus*, methicillin-susceptible | 1 (0.7) | 0 (0.0) | 0 (0.0) | 1 (0.4) |
| *Enterococcus faecalis* \| *Pseudomonas stutzeri* | 0 (0.0) | 0 (0.0) | 1 (1.2) | 1 (0.4) |
| *Enterococcus faecalis* \| *Raoultella ornithinolytica* \| *Raoultella planticola* | 1 (0.7) | 0 (0.0) | 0 (0.0) | 1 (0.4) |
| *Enterococcus faecalis* \| *Serratia marcescens* | 0 (0.0) | 0 (0.0) | 1 (1.2) | 1 (0.4) |
| *Enterococcus faecalis* \| *Staphylococcus lugdunensis* | 1 (0.7) | 0 (0.0) | 0 (0.0) | 1 (0.4) |
| *Enterococcus faecalis* \| *Streptococcus agalactiae* | 1 (0.7) | 0 (0.0) | 0 (0.0) | 1 (0.4) |
| *Enterococcus faecalis* \| *Streptococcus dysgalactiae* | 1 (0.7) | 0 (0.0) | 0 (0.0) | 1 (0.4) |
| *Enterococcus faecalis* \| Streptococcus species | 1 (0.7) | 0 (0.0) | 0 (0.0) | 1 (0.4) |
| *Enterococcus faecium* \| *Escherichia coli* \| *Klebsiella oxytoca* | 1 (0.7) | 0 (0.0) | 0 (0.0) | 1 (0.4) |
| *Enterococcus faecium* \| *Klebsiella oxytoca* | 0 (0.0) | 1 (2.9) | 0 (0.0) | 1 (0.4) |
| *Enterococcus faecium* \| *Klebsiella pneumoniae* \| *Stenotrophomonas maltophilia* | 1 (0.7) | 0 (0.0) | 0 (0.0) | 1 (0.4) |
| *Enterococcus faecium* \| Pantoea species | 0 (0.0) | 1 (2.9) | 0 (0.0) | 1 (0.4) |
| *Enterococcus faecium* \| *Proteus mirabilis* | 0 (0.0) | 1 (2.9) | 0 (0.0) | 1 (0.4) |
| *Enterococcus faecium* \| *Pseudomonas aeruginosa* | 1 (0.7) | 0 (0.0) | 0 (0.0) | 1 (0.4) |
| *Enterococcus faecium* \| *Pseudomonas aeruginosa* \| *Streptococcus pyogenes* | 1 (0.7) | 0 (0.0) | 0 (0.0) | 1 (0.4) |
| *Escherichia coli* \| *Granulicatella adiacens* | 0 (0.0) | 0 (0.0) | 1 (1.2) | 1 (0.4) |
| *Escherichia coli* \| Hafnia species \| *Streptococcus anginosus* | 0 (0.0) | 1 (2.9) | 0 (0.0) | 1 (0.4) |
| *Escherichia coli* \| *Klebsiella pneumoniae* \| *Staphylococcus aureus*, methicillin-susceptible | 1 (0.7) | 0 (0.0) | 0 (0.0) | 1 (0.4) |
| *Escherichia coli* \| *Klebsiella pneumoniae* \| *Streptococcus gallolyticus* | 1 (0.7) | 0 (0.0) | 0 (0.0) | 1 (0.4) |
| *Escherichia coli* \| *Pseudomonas aeruginosa* \| *Staphylococcus aureus*, methicillin-susceptible \| *Streptococcus mitis/oralis* | 0 (0.0) | 0 (0.0) | 1 (1.2) | 1 (0.4) |
| *Escherichia coli* \| *Raoultella ornithinolytica* \| *Staphylococcus aureus*, methicillin-resistant | 0 (0.0) | 1 (2.9) | 0 (0.0) | 1 (0.4) |
| *Escherichia coli* \| *Staphylococcus aureus*, methicillin-resistant | 0 (0.0) | 0 (0.0) | 1 (1.2) | 1 (0.4) |
| *Escherichia coli* \| *Staphylococcus aureus*, methicillin-susceptible | 1 (0.7) | 0 (0.0) | 0 (0.0) | 1 (0.4) |
| *Facklamia hominis* \| *Staphylococcus aureus*, methicillin-susceptible | 1 (0.7) | 0 (0.0) | 0 (0.0) | 1 (0.4) |
| *Fusobacterium necrophorum* \| *Gemella haemolysans* \| *Granulicatella adiacens* | 1 (0.7) | 0 (0.0) | 0 (0.0) | 1 (0.4) |
| *Fusobacterium necrophorum* \| *Streptococcus anginosus* | 0 (0.0) | 0 (0.0) | 1 (1.2) | 1 (0.4) |
| Gram negative bacilli, anaerobic \| *Serratia marcescens* \| *Staphylococcus aureus*, methicillin-resistant | 0 (0.0) | 1 (2.9) | 0 (0.0) | 1 (0.4) |
| *Granulicatella adiacens* \| *Staphylococcus aureus*, methicillin-resistant | 1 (0.7) | 0 (0.0) | 0 (0.0) | 1 (0.4) |
| *Klebsiella aerogenes* \| *Staphylococcus aureus*, methicillin-susceptible | 0 (0.0) | 1 (2.9) | 0 (0.0) | 1 (0.4) |
| *Klebsiella pneumoniae* \| *Streptococcus constellatus* | 1 (0.7) | 0 (0.0) | 0 (0.0) | 1 (0.4) |
| *Klebsiella pneumoniae* \| *Streptococcus mitis/oralis* | 0 (0.0) | 1 (2.9) | 0 (0.0) | 1 (0.4) |
| *Klebsiella pneumoniae* \| *Streptococcus salivarius* | 1 (0.7) | 0 (0.0) | 0 (0.0) | 1 (0.4) |
| *Klebsiella pneumoniae/variicola* \| *Streptococcus constellatus* | 0 (0.0) | 0 (0.0) | 1 (1.2) | 1 (0.4) |
| Methylobacterium species \| *Streptococcus pyogenes* | 0 (0.0) | 0 (0.0) | 1 (1.2) | 1 (0.4) |
| Neisseria species \| *Rothia mucilaginosa* | 0 (0.0) | 0 (0.0) | 1 (1.2) | 1 (0.4) |
| *Paeniclostridium sordellii* \| *Proteus mirabilis* | 0 (0.0) | 0 (0.0) | 1 (1.2) | 1 (0.4) |
| *Parvimonas micra* \| *Streptococcus anginosus* | 0 (0.0) | 0 (0.0) | 1 (1.2) | 1 (0.4) |
| *Parvimonas micra* \| *Streptococcus constellatus* | 1 (0.7) | 0 (0.0) | 0 (0.0) | 1 (0.4) |
| *Pasteurella multocida* \| *Streptococcus agalactiae* | 1 (0.7) | 0 (0.0) | 0 (0.0) | 1 (0.4) |
| *Prevotella bivia* \| *Streptococcus mitis/oralis* | 1 (0.7) | 0 (0.0) | 0 (0.0) | 1 (0.4) |
| *Prevotella melaninogenica* \| *Serratia marcescens* | 1 (0.7) | 0 (0.0) | 0 (0.0) | 1 (0.4) |
| *Proteus mirabilis* \| *Staphylococcus aureus*, methicillin-resistant \| *Streptococcus dysgalactiae* | 1 (0.7) | 0 (0.0) | 0 (0.0) | 1 (0.4) |
| *Proteus mirabilis* \| *Staphylococcus aureus*, methicillin-resistant \| *Streptococcus pyogenes* | 0 (0.0) | 0 (0.0) | 1 (1.2) | 1 (0.4) |
| *Proteus mirabilis* \| *Streptococcus anginosus* | 0 (0.0) | 1 (2.9) | 0 (0.0) | 1 (0.4) |
| *Proteus mirabilis* \| *Streptococcus constellatus* | 0 (0.0) | 1 (2.9) | 0 (0.0) | 1 (0.4) |
| *Proteus vulgaris* \| *Staphylococcus aureus*, methicillin-susceptible | 1 (0.7) | 0 (0.0) | 0 (0.0) | 1 (0.4) |
| *Pseudomonas aeruginosa* \| *Staphylococcus aureus*, methicillin-resistant | 1 (0.7) | 0 (0.0) | 0 (0.0) | 1 (0.4) |
| *Pseudomonas aeruginosa* \| *Streptococcus constellatus* | 0 (0.0) | 0 (0.0) | 1 (1.2) | 1 (0.4) |
| *Pseudomonas aeruginosa* \| *Streptococcus gordonii* | 0 (0.0) | 1 (2.9) | 0 (0.0) | 1 (0.4) |
| *Pseudomonas aeruginosa* \| *Veillonella dispar* | 0 (0.0) | 0 (0.0) | 1 (1.2) | 1 (0.4) |
| *Staphylococcus aureus*, methicillin-resistant \| *Staphylococcus aureus*, methicillin-susceptible \| *Streptococcus pyogenes* | 1 (0.7) | 0 (0.0) | 0 (0.0) | 1 (0.4) |
| *Staphylococcus aureus*, methicillin-resistant \| *Streptococcus anginosus* | 0 (0.0) | 0 (0.0) | 1 (1.2) | 1 (0.4) |
| *Staphylococcus aureus*, methicillin-resistant \| *Streptococcus mitis/oralis* \| *Streptococcus salivarius* | 0 (0.0) | 0 (0.0) | 1 (1.2) | 1 (0.4) |
| *Staphylococcus aureus*, methicillin-resistant \| *Streptococcus parasanguinis* | 1 (0.7) | 0 (0.0) | 0 (0.0) | 1 (0.4) |
| *Staphylococcus aureus*, methicillin-susceptible \| *Streptococcus anginosus* | 0 (0.0) | 0 (0.0) | 1 (1.2) | 1 (0.4) |
| *Staphylococcus aureus*, methicillin-susceptible \| *Streptococcus salivarius* | 1 (0.7) | 0 (0.0) | 0 (0.0) | 1 (0.4) |
| *Staphylococcus lugdunensis* \| *Streptococcus pyogenes* | 1 (0.7) | 0 (0.0) | 0 (0.0) | 1 (0.4) |

The pre-restriction, restriction, and post-restriction periods were June 26, 2023–June 25, 2024, June 26, 2024–December 23, 2024, and December 24, 2024–June 25, 2025, respectively. Data shown are the number of bloodstream infectious episodes with the listed organism(s), with percentage in parentheses reflecting this as the numerator and size of the overall group as the denominator (shown in the headings). Single-organism episodes are listed first in order from most to least frequent, followed by multi-organism episodes in order from most to least frequent.

# **Supplementary Table 12.** Effect Estimates of Single-Set Blood Culture Restriction on Episode Incidence for Primary Organism Groups.

|  | **Slope before blood culture restriction** | | **Level change upon blood culture restriction** | | **Slope during blood culture restriction** | | **Level change at end of blood culture restriction** | | **Slope after blood culture restriction** | |
| --- | --- | --- | --- | --- | --- | --- | --- | --- | --- | --- |
| **Organism group** | **IRR** | **P value** | **IRR** | **P value** | **IRR** | **P value** | **IRR** | **P value** | **IRR** | **P value** |
| All episodes | 0.998 (0.980–1.015) | P=.80 | 0.813 (0.578–1.145) | P=.24 | 0.985 (0.922–1.052) | P=.65 | 0.859 (0.530–1.391) | P=.54 | 1.051 (0.981–1.126) | P=.15 |
| Anaerobes | 1.038 (1.010–1.067) | P=.007 | 0.420 (0.161–1.093) | P=.08 | 0.995 (0.811–1.222) | P=.96 | 0.393 (0.076–2.033) | P=.27 | 1.075 (0.842–1.374) | P=.56 |
| Enterococci | 0.995 (0.958–1.033) | P=.78 | 0.697 (0.398–1.221) | P=.21 | 1.095 (1.001–1.197) | P=.05 | 0.565 (0.275–1.160) | P=.12 | 0.934 (0.852–1.023) | P=.14 |
| Fungi | 0.983 (0.896–1.079) | P=.72 | 0.813 (0.439–1.507) | P=.51 | 1.093 (0.984–1.214) | P=.10 | 0.613 (0.267–1.408) | P=.25 | 0.893 (0.810–0.985) | P=.02 |
| Gram-negative rods | 1.003 (0.980–1.027) | P=.79 | 0.952 (0.816–1.111) | P=.53 | 0.965 (0.936–0.995) | P=.02 | 1.147 (0.920–1.429) | P=.22 | 1.033 (1.005–1.061) | P=.02 |
| *Staphylococcus aureus* or *lugdunensis* | 0.998 (0.981–1.015) | P=.82 | 0.842 (0.693–1.024) | P=.09 | 1.019 (0.985–1.054) | P=.28 | 0.744 (0.590–0.938) | P=.01 | 1.002 (0.966–1.039) | P=.91 |
| Streptococci | 1.036 (0.991–1.083) | P=.12 | 0.483 (0.243–0.963) | P=.04 | 0.980 (0.880–1.092) | P=.72 | 0.434 (0.127–1.487) | P=.18 | 1.076 (0.918–1.263) | P=.37 |
| Coagulase-negative staphylococci or common commensals | 0.984 (0.964–1.004) | P=.11 | 0.989 (0.622–1.574) | P=.96 | 0.941 (0.835–1.060) | P=.32 | 1.098 (0.524–2.301) | P=.81 | 1.159 (1.027–1.308) | P=.02 |
| Polymicrobial | 0.976 (0.937–1.016) | P=.24 | 0.426 (0.098–1.853) | P=.26 | 1.083 (0.778–1.505) | P=.64 | 0.767 (0.096–6.153) | P=.80 | 0.908 (0.632–1.305) | P=.60 |

The pre-restriction, restriction, and post-restrictions periods were June 26, 2023–June 25, 2024, June 26, 2024–December 23, 2024, and December 24, 2024–June 25, 2025, respectively. We omitted showing the “Rare organism” group due to its overall rare incidence, less than 1% across all time periods.

Abbreviations: IRR, incidence rate ratio.
